# mRNA-1273 vaccination induces polyfunctional memory CD4 and CD8 T cell responses in patients with solid cancers undergoing immunotherapy or/and chemotherapy

**DOI:** 10.3389/fimmu.2024.1447555

**Published:** 2024-08-27

**Authors:** Anastasia Gangaev, Yannick van Sleen, Nicole Brandhorst, Kelly Hoefakker, Bimal Prajapati, Amrita Singh, Annemarie Boerma, Marieke van der Heiden, Sjoukje F. Oosting, Astrid A. M. van der Veldt, T. Jeroen N. Hiltermann, Corine H. GeurtsvanKessel, Anne-Marie C. Dingemans, Egbert F. Smit, Elisabeth G. E. de Vries, John B. A. G. Haanen, Pia Kvistborg, Debbie van Baarle

**Affiliations:** ^1^ Division of Molecular Oncology and Immunology, The Netherlands Cancer Institute, Amsterdam, Netherlands; ^2^ Department of Medical Microbiology and Infection Prevention, University Medical Centre Groningen, Groningen, Netherlands; ^3^ Department of Medical Oncology, University Medical Center Groningen, University of Groningen, Groningen, Netherlands; ^4^ Department of Medical Oncology and Radiology & Nuclear Medicine, Erasmus Medical Center (MC)-Cancer Institute, Rotterdam, Netherlands; ^5^ Department of Pulmonary Diseases, University Medical Centre Groningen, Groningen, Netherlands; ^6^ Department of Viroscience, Erasmus Medical Center (MC) Cancer Institute, University Medical Centre, Rotterdam, Netherlands; ^7^ Department of Respiratory Medicine, Erasmus Medical Centre, Rotterdam, Netherlands; ^8^ Department of Thoracic Oncology, The Netherlands Cancer Institute, Amsterdam, Netherlands; ^9^ Centre for Infectious Disease Control, National Institute for Public Health and the Environment, Utrecht, Netherlands

**Keywords:** SARS-CoV-2-specific T cells, COVID-19, cancer, immunotherapy, chemotherapy, COVID-19 vaccination, chemoimmunotherapy

## Abstract

**Introduction:**

Research has confirmed the safety and comparable seroconversion rates following SARS-CoV-2 vaccination in patients with solid cancers. However, the impact of cancer treatment on vaccine-induced T cell responses remains poorly understood.

**Methods:**

In this study, we expand on previous findings within the VOICE trial by evaluating the functional and phenotypic composition of mRNA-1273-induced T cell responses in patients with solid tumors undergoing immunotherapy, chemotherapy, or both, compared to individuals without cancer. We conducted an ELISpot analysis on 386 participants to assess spike-specific T cell responses 28 days after full vaccination. Further in-depth characterization of using flow cytometry was performed on a subset of 63 participants to analyze the functional phenotype and differentiation state of spike-specific T cell responses.

**Results:**

ELISpot analysis showed robust induction of spike-specific T cell responses across all treatment groups, with response rates ranging from 75% to 80%. Flow cytometry analysis revealed a distinctive cytokine production pattern across cohorts, with CD4 T cells producing IFNγ, TNF, and IL-2, and CD8 T cells producing IFNγ, TNF, and CCL4. Variations were observed in the proportion of monofunctional CD4 T cells producing TNF, particularly higher in individuals without cancer and patients treated with chemotherapy alone, while those treated with immunotherapy or chemoimmunotherapy predominantly produced IFNγ. Despite these differences, polyfunctional spike-specific memory CD4 and CD8 T cell responses were comparable across cohorts. Notably, immunotherapy-treated patients exhibited an expansion of spike-specific CD4 T cells with a terminally differentiated effector memory phenotype.

**Discussion:**

These findings demonstrate that systemic treatment in patients with solid tumors does not compromise the quality of polyfunctional mRNA-1273-induced T cell responses. This underscores the importance of COVID-19 vaccination in patients with solid cancers undergoing systemic treatment.

## Introduction

The global healthcare system was challenged unprecedentedly during the COVID-19 pandemic, leading to over 774 million diagnosed cases and more than 7 million deaths from COVID-19 by March 2024 (1). Individuals with cancer faced an elevated risk of COVID-19 morbidity and mortality (2). Therefore, leading oncology organizations, including ASCO, ESMO, AACR, and SITC, have recommended COVID-19 vaccination for patients with cancer early in the pandemic despite their exclusion from pivotal registration studies (3–6). Subsequent research has demonstrated the safety (7–10), and comparable seroconversion rates in patients with solid tumors to those observed in the general population (11–16).

While assessments of virus-specific immune defense primarily focus on the humoral response, T cell immunity is crucial for the successful control of infection and prevention of disease (17). Furthermore, T cells are less affected by antigenic drifts compared to humoral responses (18–22). Nevertheless, comprehensive characterization of COVID-19 vaccine-induced T cell responses has predominantly focused on large trial cohorts that exclude individuals with diseases such as cancer (18, 19, 21, 23–26). Cytokine or activation-induced marker assays have been used to assess COVID-19 vaccine-induced T cell responses in patients with cancer (11, 16, 22, 27–30), however, phenotypic and functional characterization in this population remains limited. More importantly, a critical knowledge gap persists regarding the impact of systemic cancer treatments on the ability to mount effective T-cell responses to COVID-19 vaccination.

To improve our understanding of humoral and cellular COVID-19 vaccine-induced immune responses in patients with solid cancers undergoing systemic cancer treatment, we initiated the VOICE study (‘vaccination against COVID in cancer’) (31). This prospective, national, multicenter, and multi-cohort study allows the comparative analysis of vaccine-induced immune responses in a control cohort comprising individuals without cancer (CTRL), and patients with solid tumors undergoing immunotherapy (IT), chemotherapy (CT) or chemoimmunotherapy (CT/IT). First, consistent with previous studies (7–10, 32), our findings confirmed the clinical safety of COVID-19 vaccination in patients with cancer, as well as adequate seroconversion rates and T cell responses irrespective of treatment modality (33). Additionally, we have shown durable T cell responses up to 1 year after vaccination and demonstrated that the time since the last vaccination dose, but not the cancer treatment, was a risk factor for impaired antibody responses (34). Third, we have demonstrated that booster vaccination in patients with cancer was safe and effective in increasing humoral immune responses against wild-type SARS-CoV-2 but not Omicron (35). What remains unclear, however, is whether T cell responses post-vaccination in patients with solid cancer are functionally equivalent to those in individuals without cancer and how the treatment may alter the phenotype of vaccine-specific T cells. In this study, we build on our previous findings within the VOICE trial and provide further insights into the functional and phenotypic characteristics of vaccine-induced spike-specific CD4 and CD8 T cell responses.

## Results

### Identification of spike-specific T cell responses

**Figure 1:**
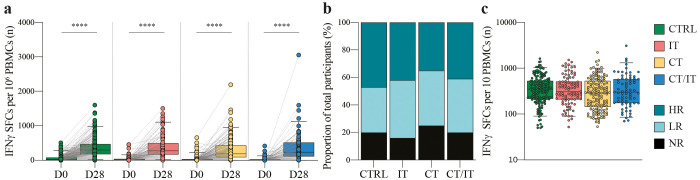
Global characterization of the spike-specific T cell response using ELISpot. **(A)** Spike-specific T cell responses measured before and 28 days after the second vaccination in CTRL (n = 214), and patients with cancer treated with IT (n = 104), CT (n = 177), and CT/IT (n = 87). Box plots indicate the median (line), 25th and 75th percentile (box), 5th and 95th percentile (whiskers), and individual data points (single circles). Statistical significance between time points for each cohort was tested with a two-tailed Wilcoxon signed-rank test; *** *P* < 0.0001. **(B)** Proportion of non-responders and responders. **(C)** Spike-specific T cell responses measured 28 days after the second vaccination in CTRL (n = 170), and patients with cancer treated with IT (n = 87), CT (n = 132), and CT/IT (n = 70) identified as responders. Box plots indicate the median (line), 25th and 75th percentile (box), 5th and 95th percentile (whiskers), and individual data points (single circles). Statistical significance between cohorts was tested with a non-parametric Kruskal-Wallis and Dunn’s multiple comparison test; no significance was found. SFC: spot-forming cells, D0: day 0, D28: day 28, CTRL, control; IT, immunotherapy; CT, chemotherapy; CT/IT, chemoimmunotherapy; HR, high responder; LR, low responder; NR, non-responder.

We first assessed the global induction of spike-specific T cell responses in PBMC samples collected at baseline and 28 days after the second mRNA-1273 vaccine dose. ELISpot data analysis of 582 participants (CTRL n = 214, IT n = 104, CT n = 177, CT/IT n = 87, **Table 1**) showed a significant induction of spike-specific T cells in all analyzed cohorts (**Figure 1A**). A total of 469 participants were identified as vaccine responders, and the proportion of responders was similar across cohorts (CTRL = 80%, IT = 84%, CT = 75%, CT/IT = 80%, **Figure 1B**). Overall, no notable differences were observed between ELISpot responders and non-responders (**Supplementary Table 1**). Notably, the treatment intent of all non-responders treated with IT alone was non-curative, while responders in this cohort included both curative and non-curative cases, highlighting a better WHO performance status among responders treated with IT. Further analysis of responders showed no significant differences in the frequency of spike-specific T cells between the analyzed cohorts (**Figure 1C**). In line with our previous findings (33–35), the mRNA-1273 vaccine was effective in inducing spike-specific T cell responses in all cohorts.

**Table 1:** Demographic and clinical characteristics of individual cohorts analyzed by ELISpot.

	CTRL	IT	CT	CT/IT
	Individuals without cancer	Patients treated with Immunotherapy	Patients treated with Chemotherapy	Patients treated with Chemo-Immunotherapy
**Total number of participants included in data analysis (n)**	214	104	177	87
**Age, median (range)**	61 (20-87)	66 (29-83)	59 (19-76)	62 (33-82)
Gender, n (%)				
Female	103 (48%)	35 (34%)	109 (62%)	46 (53%)
Male	111 (52%)	69 (66%)	68 (38%)	41 (47%)
WHO performance status, n (%)				
0	199 (93%)	73 (70%)	104 (59%)	36 (41%)
1	14 (6.5%)	31 (30%)	70 (40%)	43 (49%)
2	0 (0%)	0 (0%)	3 (2%)	5 (6%)
Unknown	1 (0.5%)	0 (0%)	0 (0%)	0 (0%)
Tumor stage, n (%)				
I	n/a	2 (2%)	13 (7%)	0 (0%)
II	n/a	1 (1%)	31 (18%)	0 (0%)
III	n/a	27 (26%)	39 (22%)	7 (8%)
IV	n/a	74 (71%)	93 (53%)	80 (92%)
Unknown	n/a	0 (0%)	1 (1%)	0 (0%)
Treatment intent, n (%)				
Curative	n/a	39 (38%)	96 (54%)	12 (14%)
Non-curative	n/a	65 (63%)	81 (46%)	75 (86%)
Primary tumor localization, n (%)				
Bone, articular cartilage, and soft tissues	n/a	1 (1%)	7 (4%)	0 (0%)
Breast	n/a	0 (0%)	56 (32%)	0 (0%)
Central nervous system	n/a	0 (0%)	7 (4%)	0 (0%)
Digestive tract	n/a	4 (4%)	51 (29%)	0 (0%)
Endocrine glands	n/a	0 (0%)	3 (2%)	0 (0%)
Female genital organs	n/a	0 (0%)	15 (8%)	0 (0%)
Head and neck	n/a	1 (1%)	5 (3%)	1 (1%)
Male genital organs	n/a	0 (0%)	12 (7%)	0 (0%)
Other/unspecified sites	n/a	1 (1%)	0 (0%)	0 (0%)
Respiratory tract	n/a	18 (17%)	12 (7%)	86 (99%)
Skin	n/a	55 (53%)	0 (0%)	0 (0%)
Urinary tract	n/a	24 (23%)	8 (5%)	0 (0%)

Detailed information for individual participants is provided in **Supplementary Data Sheet 1**.

### Kinetics of spike-specific CD4 and CD8 T cell responses

**Figure 2:**
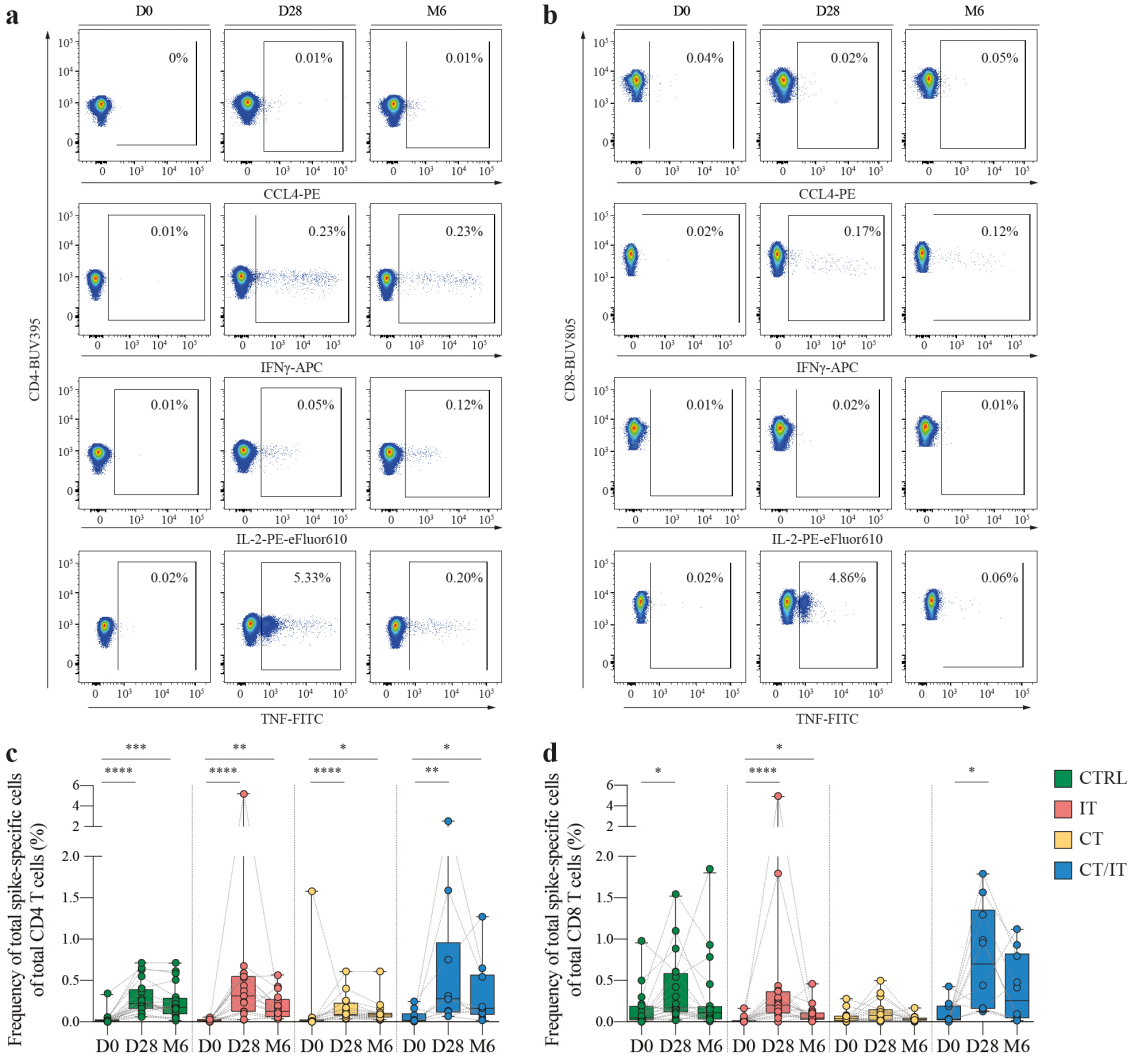
Kinetics of the spike-specific CD4 and CD8 T cell responses. Representative flow cytometry plots illustrating the production of CCL4, IFNγ, IL-2 and TNF in **(A)** CD4 and **(B)** CD8 T cells measured before, 28 days and 6 months after the second vaccination. Percentages represent the frequency of cytokine producing cells of total CD4 or CD8 T cells. Full gating strategy is shown in **Supplementary Figure 2**. Kinetics of spike-specific **(C)** CD4 and **(D)** CD8 T cell responses measured by flow cytometry before, 28 days and 6 months after the second vaccination in CTRL (n = 20), and patients with cancer treated with IT (n = 17), CT (n = 16), and CT/IT (n = 10). Spike-specific CD4 or CD8 T cells were defined based on production of CCL4, IFNγ, IL-2 and/or TNF. Box plots indicate the median (line), 25th and 75th percentile (box), 5th and 95th percentile (whiskers), and individual points (single circles). Statistical significance between time points was tested with a non-parametric Friedman and Dunn’s multiple comparison test for each cohort; * *P* < 0.05; ** *P* < 0.01; *** *P* < 0.001; **** *P* < 0.0001. D0: day 0, D28: day 28, CTRL, control; IT, immunotherapy; CT, chemotherapy; CT/IT, chemoimmunotherapy.

For further in-depth characterization of CD4 and CD8 T cell responses using flow cytometry, a subset of 63 ELISpot responders with sufficient material (CTRL n = 20, IT n = 17, CT n = 16, CT/IT n = 10, **Table 2**) was randomly selected. PBMC samples collected at baseline, 28 days, and 6 months after vaccination were analyzed to profile the induction and monitor changes in the magnitude of the spike-specific T cell response over time. A total of 4 cytokines (CCL4, IFNγ, IL-2, and TNF) were measured to detect a broad range of functional spike-specific CD4 and CD8 T cell responses (**Figures 2A, B**, **Supplementary Figure 2**). The combined frequency of IFNγ-producing CD4 and CD8 T cells detected by flow cytometry and the number of IFNγ-SFCs measured by ELISpot detected 28 days after vaccination correlated (Spearman r = 0.56, p < 0.0001, **Supplementary Figure 3A**).

**Table 2:** Demographic and clinical characteristics of individual cohorts analyzed by flow cytometry.

	CTRL	IT	CT	CT/IT
	Individuals without cancer	Patients with cancer treated with Immunotherapy	Patients with cancer treated with Chemotherapy	Patients with cancer treated with Chemo-Immunotherapy
**Total number of participants included in data analysis (n)**	20	17	16	10
**Age, median (range)**	65 (41-76)	62 (39-82)	57 (19-73)	57 (33-79)
Gender, n (%)				
Female	12 (60%)	11 (65%)	2 (13%)	6 (60%)
Male	8 (40%)	6 (35%)	14 (88%)	4 (40%)
WHO performance status, n (%)				
0	17 (85%)	15 (88%)	15 (94%)	4 (40%)
1	3 (15%)	2 (12%)	2 (13%)	5 (50%)
2	0 (0%)	0 (0%)	0 (0%)	1 (10%)
Tumor stage, n (%)				
II	n/a	0 (0%)	3 (19%)	0 (0%)
III	n/a	3 (18%)	4 (25%)	1 (10%)
IV	n/a	14 (82%)	8 (50%)	9 (90%)
Unknown	n/a	0 (0%)	1 (6%)	0 (0%)
Treatment intent, n (%)				
Curative	n/a	3 (18%)	8 (50%)	1 (10%)
Non-curative	n/a	14 (82%)	8 (50%)	9 (90%)
Primary tumor localization, n (%)				
Breast	n/a	0 (0%)	3 (19%)	0 (0%)
Central nervous system	n/a	0 (0%)	2 (13%)	0 (0%)
Digestive tract	n/a	0 (0%)	6 (38%)	0 (0%)
Male genital organs	n/a	0 (0%)	1 (6%)	0 (0%)
Respiratory tract	n/a	6 (35%)	3 (19%)	10 (100%)
Skin	n/a	7 (41%)	0 (0%)	0 (0%)
Urinary tract	n/a	4 (24%)	1 (6%)	0 (0%)

Detailed information for individual participants is provided in **Supplementary Data Sheet 1**.

The baseline magnitude of spike-specific CD4 and CD8 T cell responses identified by flow cytometry based on production of CCL4, IFNγ, IL-2, and/or TNF ranged between 0.01% and 0.05% (CD4: CTRL = 0.02%, IT = 0.01%, CT = 0.01%, CT/IT = 0.02%, **Figure 2C** and CD8: CTRL = 0.05%, IT = 0.01%, CT = 0.04%, CT/IT = 0.03%, **Figure 2D**). In some participants, baseline spike-specific CD4 and CD8 T cell responses reached up to 1.75% of total CD4/CD8 T cells suggesting the presence of pre-existing T cell immunity elicited by other previous circulating coronaviruses as demonstrated by various studies outlined in this review (36). An increase in the median magnitude of spike-specific CD4 and CD8 T cells 28 days after vaccination was found in all cohorts. The majority of the spike-specific T cell response 28 days after vaccination consisted of CD4 T cells (median proportion: CTRL = 57%, IT = 56% CT = 66%), while in CT/IT-treated patients an equal proportion of spike-specific CD4 and CD8 T cells was found (**Supplementary Figure 3B**). The proportion of CD4 T cells increased 6 months after vaccination.

The median magnitude of vaccine-induced CD4 T cell responses (CTRL = 0.22%, IT = 0.31%, CT = 0.09%, CT/IT = 0.31%) and CD8 T cell responses (CTRL = 0.17%, IT = 0.20%, CT = 0.08%, CT/IT = 0.70%) was lowest in CT-treated patients. Notably, in contrast to the ELISpot data analysis, the frequency of the total spike-specific CD4 and CD8 T cell response combined detected by flow cytometry 28 days after vaccination was lower in CT-treated patients compared to CTRLs and CT/IT-treated patients (**Supplementary Figure 3C**). However, it is important to note that these results likely reflect a selection bias due to the unequal distribution of low and high responders selected for flow cytometry analysis compared to the original cohort analyzed by ELISpot (**Supplementary Figures 3D, E**).

An overall decrease in the magnitude of spike-specific CD4 and CD8 T cell responses was observed 6 months after vaccination. Specifically, spike-specific CD4 T cell responses declined in CTRLs, and patients treated with IT and CT/IT, but not in CT-treated patients (median fold-change decrease: CTRL = 1.3, CT = 1, IT = 2.5, CT/IT = 1.7). Spike-specific CD8 T cell responses consistently decreased in all cohorts (median fold-change decrease: CTRL = 1.6, IT = 4.3, CT = 2.6, CT/IT = 2.7). Overall, these findings demonstrate the induction of spike-specific CD4 and CD8 T cell responses upon vaccination and a consistent decrease of spike-specific T cell responses 6 months after vaccination.

### Functional composition of spike-specific CD4 and CD8 T cell responses

**Figure 3:**
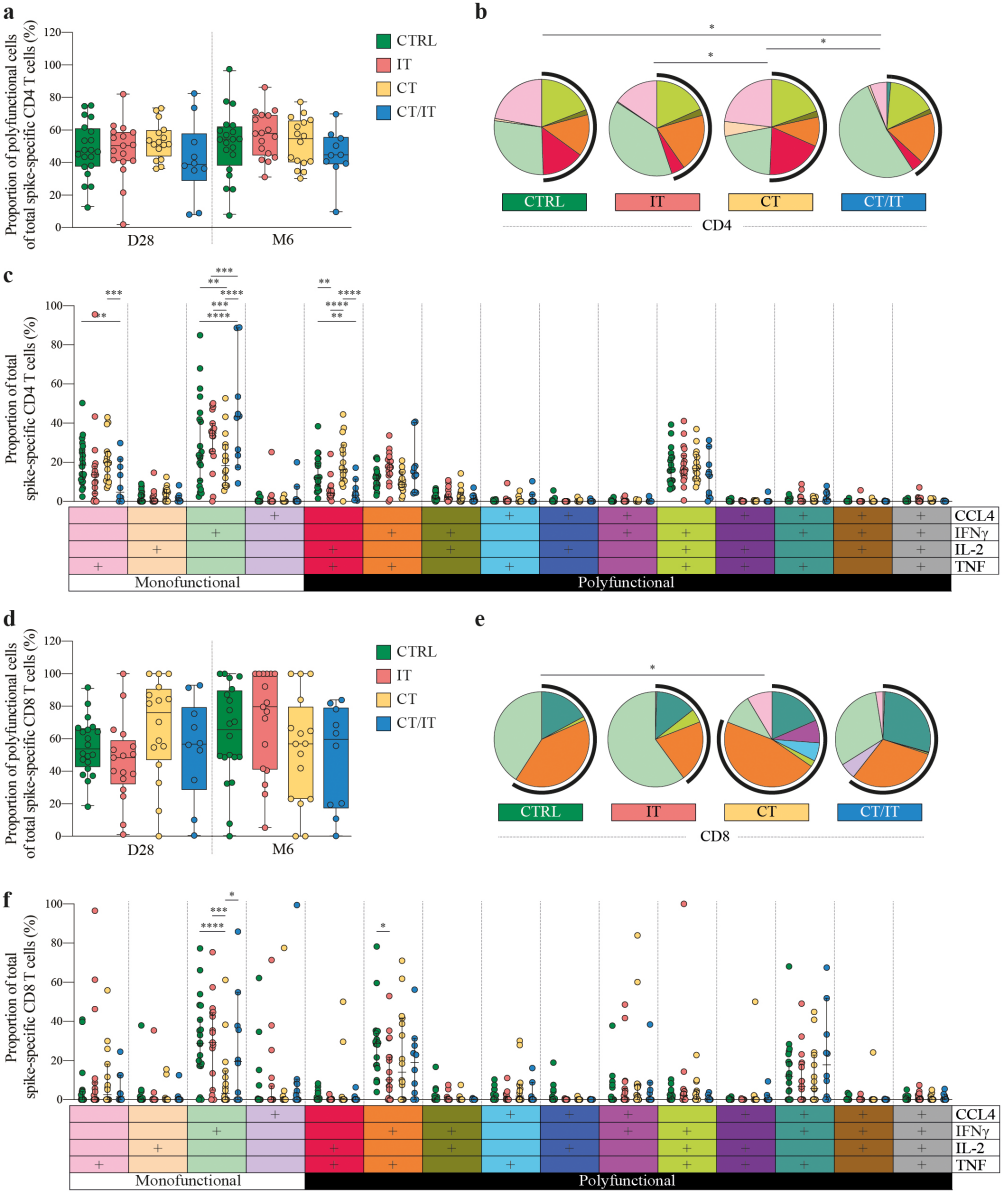
Functional quality of the spike-specific CD4 and CD8 T cell response in CTRL (n = 20), and patients with cancer treated with IT (n = 17), CT (n = 16), and CT/IT (n = 10). Proportion of polyfunctional spike-specific T cells of the total spike-specific **(A)** CD4 and **(D)** CD8 T cell response 28 days and 6 months after the second vaccination. Box plots indicate the median (line), 25th and 75th percentile (box), 5th and 95th percentile (whiskers), and individual points (single circles). Statistical significance between cohorts for each time point was tested with a non-parametric Kruskal-Wallis and Dunn’s multiple comparison test; no significance was found. SPICE analysis of spike-specific **(B)** CD4 and **(E)** CD8 T cell responses 28 days after the second vaccination. Pie charts represent the median proportion of individual spike-specific T cell populations of the total spike-specific CD4 or CD8 T cell response. Pie charts represent the cytokine (co-)production patterns of individual spike-specific T cell populations. Arc charts indicate the proportion of polyfunctional spike-specific T cells of the total spike-specific CD4 or CD8 T cell response. Individual pie chart and arc colors legends are indicated in **(C, F)**. Statistical significance between cohorts was tested with a permutation test with a multiple comparison test of 10.000 iterations. Proportion of individual spike-specific T cell populations producing CCL4-, IFNγ-, IL-2- and/or TNF of the total spike-specific **(C)** CD4 and **(F)** CD8 T cell response. Median (middle line), 95% confidence interval (whiskers) and individual points (single circles) are shown. Statistical significance between cohorts was tested with an ordinary two-way ANOVA Tukey’s multiple comparisons test. * *P* < 0.05, ** *P* < 0.01, *** *P* < 0.001, **** *P* < 0.0001. D0: day 0, D28: day 28, CTRL, control; IT, immunotherapy; CT, chemotherapy; CT/IT, chemoimmunotherapy.

Stimulation with peptide pools from the SARS-CoV-2 spike protein revealed distinct cytokine production patterns, with spike-specific CD4 T cells primarily producing IFNγ, TNF, and IL-2 (**Supplementary Figure 4A**), while spike-specific CD8 T cells predominantly produced IFNγ, TNF, and CCL4 (**Supplementary Figure 4B**). Notably, the technical control using phorbol 12-myristate 13-acetate and ionomycin demonstrated a robust production of all four cytokines (CCL4, IFNγ, IL-2, and/or TNF) in both CD4 and CD8 T cells (**Supplementary Figure 5**). To further assess the functional quality of spike-specific responses, we focused our analysis on the proportion of CCL4, IFNγ, IL-2, and/or TNF-producing cells within the total spike-specific response that was not influenced by the previously identified selection bias, rather than the frequency of total CD4 or CD8 T cells (**Supplementary Figures 5C, D**). The proportion of the total polyfunctional spike-specific CD4 T cell response was similar between cohorts (**Figure 3A**) and analyzed time points after vaccination (**Supplementary Figure 6A**). The functional composition of spike-specific CD4 T cell responses varied between cohorts (**Figure 3B** and **Supplementary Figure 6B**). These differences were primarily attributed to variations in the production of monofunctional CD4 T cells, with a higher proportion of monofunctional cells producing TNF in CTRLs and CT-treated patients, while the majority of monofunctional cells in IT- and CT/IT-treated produced IFNγ (**Figure 3C**). Monofunctional CD4 T cells producing IFNγ were lower in all cohorts 6 months after vaccination than day 28 (**Supplementary Figure 7**). Additionally, we observed differences between cohorts in the proportion of double cytokine-producing CD4 T cells producing IL-2 and TNF that was higher in CTRL and CT-treated patients compared to patients treated with IT and CT/IT. Notably, IL-2 and TNF-producing CD4 T cells decreased in CTRLs and CT-treated patients 6 months after vaccination. However, the proportion of triple cytokine-producing CD4 T cells producing IFNγ, IL-2, and TNF remained stable 28 days and 6 months after vaccination for all cohorts.

Similar to the CD4 T cell response, the overall proportion of polyfunctional cells of the total CD8 T cell response did not differ across cohorts (**Figure 3D**). Nevertheless, IT-treated patients showed an increase in the proportion of polyfunctional CD8 T cells at 6 months compared to day 28 (**Supplementary Figure 8A**). We observed differences in the overall functional composition of the spike-specific CD8 T cell response between CTRLs and CT-treated patients 28 days after vaccination (**Figure 3E**), and a lower proportion of monofunctional CD8 T cells producing IFNγ in patients treated with CT alone (**Figure 3F**). However, these differences were not sustained 6 months after vaccination (**Supplementary Figure 8B**). Notably, the increase in the proportion of polyfunctional CD8 T cells in IT-treated was primarily associated with a reduction in the monofunctional population producing IFNγ (**Supplementary Figure 9**). The proportion of double cytokine-producing CD8 T cells producing IFNγ and TNF was highest in CTRLs, however, the proportion of triple cytokine-producing cells producing CCL-4, IFNγ, and TNF was similar across cohorts. In summary, the proportion of the triple cytokine-producing cells within the spike-specific CD4 and CD8 T cell response remained consistent across all cohorts, indicating similar levels of protection that persisted up to 6 months after vaccination.

### Phenotype of spike-specific CD4 and CD8 T cell responses

**Figure 4:**
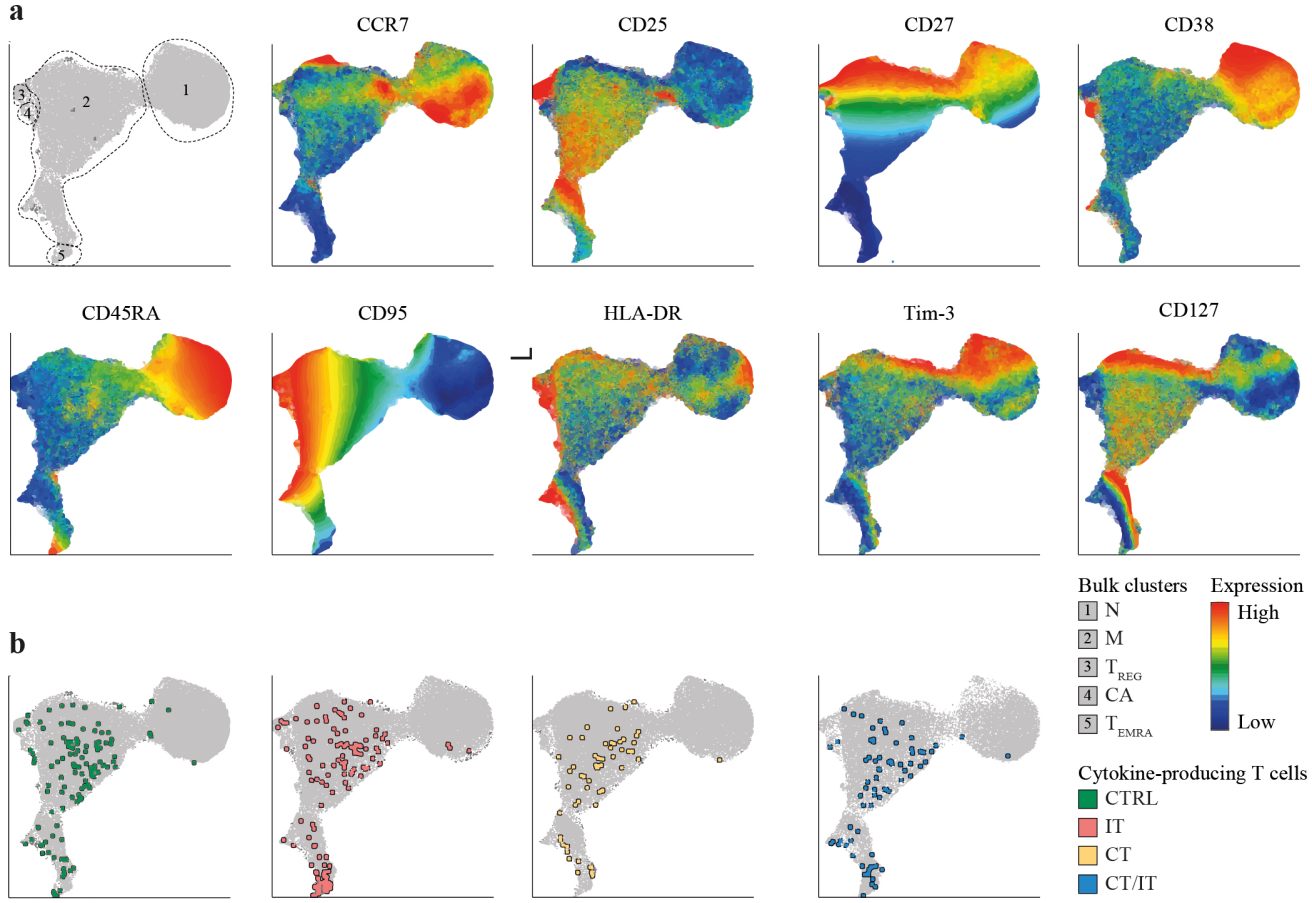
Phenotype of the spike-specific CD4 T cell response 28 days after the second vaccination. UMAP plots were created based on merged files containing both DMSO-treated and spike-specific CD4 T cells of each participant (total participants n = 63, cells per sample n = 5,000). **(A)** UMAP depicting identified clusters and scaled production of analyzed markers. **(B)** UMAP depicting the localization of polyfunctional spike-specific CD4 T cells in each cohort. Polyfunctional spike-specific CD4 T cells were defined as cells producing two or more of the analyzed cytokines (TNF, IFNγ, IL-2). UMAP, Uniform Manifold Approximation and Projection; N, naïve; M, memory; T_REG_, regulatory T cell; CA, chronically activated; T_EMRA_, terminally differentiated effector memory T cell; CTRL, control; IT, immunotherapy; CT, chemotherapy; CT/IT, chemoimmunotherapy.

To further explore the phenotype of identified spike-specific CD4 and CD8 T cell responses between cohorts, we made use of UMAP plots. We included both, spike-specific CD4 or CD8 T cells (identified based on production of CCL4, IFNγ, IL-2, and TNF) and bulk T cells in our analysis to establish a reference point for delineating the phenotype of spike-specific T cells within the overall population 28 days after vaccination. UMAP analysis of CD4 T cells revealed naïve cells (cluster 1: CD45RA^+^, CCR7^+^, CD38^+^ and CD95^-^) and several memory clusters including a large heterogenous population (cluster 2: CCR7^low/high^, CD25^low/high^, CD27^low/high^), and three smaller populations with a regulatory (cluster 3: CD25^high^CD127^-^), a ‘chronically activated’ (cluster 4: CD38^high^, HLA-DR^+^), and a T_EMRA_ (cluster 5: CD45RA^int/high^, CCR7^-^, CD27^-^) phenotype (**Figure 4A**).

We next explored the localization of spike-specific CD4 T cells within the identified CD4 T cell clusters. Nonspecific cytokine production was observed in a minor proportion of unstimulated CD27^-^, CCR7^-^ memory CD4 T cells (**Supplementary Figure S10A**). The vast majority of monofunctional (**Supplementary Figure 10B**) and polyfunctional (**Figure 4B**) spike-specific CD27^low^, CCR7^low^, CD25^int^ CD4 T cells were located within memory cluster 2 and showed no discernible phenotypical differences. Both regulatory (cluster 3) and ‘chronically activated’ (cluster 4) cells did not substantially contribute to the pool of spike-specific CD4 T cells. Notably, a substantial proportion of polyfunctional spike-specific CD4 T cells in patients treated with IT and CT/IT were located in cluster 5 (**Supplementary Figure 10C**).

UMAP representation of CD8 T cells revealed naïve cells (cluster 1: CD45RA^+^, CCR7^+^, CD38^+^, and CD95^-^) and several memory clusters including a large memory population (cluster 2), and smaller subpopulations with a T_EMRA_ (cluster 3: CD45RA^int/high^, CCR7^-^, CD27^-^), ‘innate-like’ (cluster 4: NKG2A^+^), and ‘chronically activated’ (cluster 5: CD38^high^, HLA-DR^+^) phenotype (**Supplementary Figure 11A**). Patients treated with IT and CT/IT exhibited a lower proportion of naïve cells compared to CTRLs and CT-treated patients (**Supplementary Figure 11B**). We observed spontaneous cytokine production in unstimulated CD8 T cells that were polyfunctional and predominantly located in cluster 3, indicative of a typical T_EMRA_ phenotype (**Supplementary Figure 11C**). Polyfunctional CD8 T cells were, however, more frequent and although a substantial proportion of these cells mirrored the phenotype observed in unstimulated samples, spike-stimulated (but not unstimulated) CD8 T cells were present in memory cluster 2 (**Supplementary Figures 11B and D**). ‘Innate-like’ CD8 T cells were found in both, unstimulated and spike-stimulated samples; however, these cells showed a tendency to be expanded in patients undergoing CT/IT. Overall, these results show a robust induction of polyfunctional memory spike-specific CD4 and CD8 T cells in all cohorts while IT-treated patients display expansion of spike-specific CD4 T cells with a T_EMRA_ phenotype.

## Discussion

Consistent with our previous reports (33–35), we demonstrate a robust induction of vaccine-induced spike-specific T cell responses in patients with solid tumors irrespective of cancer treatment. Through a comprehensive in-depth analysis of spike-specific T cell responses, we expand on these findings and show distinct cytokine production patterns, with CD4 T cells primarily producing IFNγ, TNF, and IL-2, while CD8 T cells predominantly produce IFNγ, TNF, and CCL4. Furthermore, we show cohort-specific variations in monofunctional spike-specific CD4 T cells. While individuals without cancer and patients with cancer treated with CT alone harbor monofunctional spike-specific CD4 T cell producing TNF, the majority of monofunctional CD4 T cells in patients treated with IT alone or in combination with CT produce IFNγ. Lastly, and more importantly, we show a robust induction of polyfunctional memory spike-specific T cell responses in patients with cancer regardless of treatment despite a predictable decrease in response frequency 6 months after vaccination.

The functional characteristics of T cells play a crucial role in clinical and immunological functions, with polyfunctional cells linked to heightened protection (37–39). Our findings show that cancer treatment in patients with solid tumors does not compromise the quality of mRNA-1273-induced CD4 and CD8 T cell responses, aligning closely with responses observed in the general population (18). Previous studies have reported adequate humoral (2, 7, 40) and cellular (11) immune responses in patients treated with IT. Furthermore, acute SARS-CoV-2 infections in patients with cancer treated with IT was not associated with severe disease and has been suggested to be beneficial in accelerating and amplifying long-term T cell immunity (41). The compromised epithelial barrier integrity in individuals undergoing chemotherapy, however, has been associated with an increased risk of microbial translocation and infection (42). Impaired COVID-19 vaccine-induced T cell responses have indeed been reported in patients with solid tumors undergoing chemotherapy (28) and recently chemotherapy has emerged as a risk factor for impaired humoral responses (22). CT has also been associated with an increased risk of breakthrough infections supporting the role of vaccine boosters (13).

SARS-CoV-2 breakthrough infections are increasingly common as antibody titers decline over time, most specifically at the mucosa surfaces; a trend further complicated by the continuous emergence of viral variants. Our findings highlight that the majority of polyfunctional spike-specific CD4 and CD8 T cells exhibit a memory phenotype in all cohorts irrespective of treatment, suggesting that vaccine-induced T cell memory likely supports the re-activation of T follicular helper (Tfh) cells that play a critical role in B cell proliferation and activation through CD40L signaling and cytokine release, thereby driving humoral immunity (43). Patients with lung cancer undergoing IT alone have been shown to acquire an exhausted CD4 T cell phenotype based on expression of inhibitory receptors (30). In contrast, influenza vaccination in patients receiving PD-1 blockade displayed a more robust follicular helper CD4 T cell response compared to patients without treatment (44). Our data also show treatment-specific effects, such as the expansion of CD4 T cells with a terminally differentiated phenotype (memory cells expressing CD45RA) and a decreased proportion of naïve T cells in patients treated with IT. Furthermore, the substantial presence of monofunctional spike-specific CD4 T cells producing IFNγ in our study, notably in patients treated with IT alone or in combination with CT, raises potential concerns. These cells have been associated with limited capacity for sustained memory and represent the final stage of CD4 T cell differentiation (45–47). Given the small sample size in our cohort, future studies are warranted to comprehensively understand the long-term impact of cancer treatments, particularly IT, on the quality of vaccine-induced T cell responses in cancer patients.

The VOICE study reports on the humoral and cellular immunity upon vaccination with mRNA-1273, however, several questions remain to be addressed in future studies. First, it is important to note, that differences in seroconversion rates have been reported between different types of COVID-19 vaccines in patients with cancer (7, 48, 49). Understanding the impact of individual vaccines on T cell quality is limited, with one study indicating that mRNA-1273 vaccination induced the largest proportion of polyfunctional T cells compared to other COVID-19 vaccines in the general population (21). Second, the flow cytometry analysis in our study is constrained by a small sample size, especially in patients treated with CT/IT, highlighting the need for future investigations in larger cohorts. Third, our study cohorts reflect treatment-related differences in primary tumor localization. Although it is unlikely that the primary tumor localization significantly impacts the quality of vaccine-induced T cell responses due to the vast majority of patients having stage IV cancer, our study cannot draw definitive conclusions regarding distinct solid tumor types.

In summary, our study provides important insights into T cell responses following mRNA-1273 vaccination in patients receiving systemic cancer treatment, and further support COVID-19 vaccination in patients with solid tumors. A deeper understanding of the role of T cell immunity for protection against SARS-CoV-2 infection and disease should provide a foundation for improving the use of current vaccines and the development of next-generation vaccines.

## Materials and methods

### Patient material

Peripheral blood mononuclear cell (PBMC) samples were obtained within the previously published VOICE trial (ClinicalTrials.gov identifier, NCT04715438) (31, 33–35). In brief, the VOICE trial is an investigator-initiated, prospective, non-inferiority trial conducted at three centers in the Netherlands: University Medical Centre in Groningen, Erasmus Medical Centre in Rotterdam, and the Netherlands Cancer Institute in Amsterdam. A total of 791 participants without prior or current confirmed SARS-CoV-2 infection were enrolled into four cohorts: CTRL (individuals without cancer, n = 247), and patients with solid tumors, regardless of stage and histology, who were treated with IT (single-agent monoclonal antibody against PD-1 or PD-L1, n = 137), CT (any type or combination of cytotoxic chemotherapy, n = 244), or CT/IT (cytotoxic chemotherapy in combination with immunotherapy, n = 163). The most recent immunotherapy administration had to be within 3 months and the most recent chemotherapy administration within 4 weeks before vaccination. All participants received two doses of the mRNA-1273 vaccine 28 days apart. PBMCs samples were collected immediately after blood withdrawal before the first vaccination, as well as 28 days and 6 months after the second vaccination. An overview of all patient characteristics and a flow diagram indicating the selection steps for individual analyses conducted in this study are shown in **Supplementary Data Sheet 1** and **Supplementary Figure 1**, respectively. All participants provided written, informed consent and the trial was done in accordance with the principles of the Declaration of Helsinki, Good Clinical Practice guidelines, and applicable government regulations. The trial protocol was approved by the medical ethics committee of the University Medical Centre Groningen.

### ELISpot assay

MultiScreen HTS IP filter plates (Millipore, MSIPS4510) were activated with ethanol (35% v/v) and coated with anti-human IFNγ antibody (Mabtech, 3420-3-250, 5 μg/mL) overnight at 4°C. The plates were blocked with X-VIVO (Lonza, BE02-060F) medium supplemented with human serum (Sigma, H6914, 2% v/v) for 1 hour at 37°C. PBMCs were thawed, washed twice, and incubated at 37°C for 60 minutes in X-VIVO medium supplemented with human serum (2% v/v). A total of 2x10^5^ PBMCs were stimulated for 20 hours at 37°C with 2 15-mer peptide pools derived from the SARS-CoV-2 spike protein (JPT Peptide Technologies, 0.5 μg/mL). All stimulations were executed in triplicates. Equimolar amounts of DMSO (Sigma Aldrich, 276855) and phytohemagglutinin (PHA, Remel Europe Ltd, HA16, 4 μg/mL) were used as negative and positive control, respectively. After incubation, ELISpot plates were washed with phosphate-buffered saline supplemented with Tween 20 (0.05% v/v). Anti-human biotinylated IFNγ antibody (Mabtech, 3420-6-250, 1:1000) and poly-HRP buffer (ThermoFisher, N500, 0.05% v/v) were added to phosphate-buffered saline for 1.5 hours. At room temperature, washing was repeated, followed by the addition of streptavidin poly-HRP (Sanquin, M2051, 1:6000) in poly-HRP buffer for 1 hour. After the final wash, spot forming cells (SFCs) were visualized using 3,3’,5,5’-tetramethylbenzidine substrate (Mabtech, 3651-10) according to the manufacturer’s protocol. SFCs were quantified with the AID ELISpot/Fluorospot reader and expressed as SFCs/10^6^ PBMCs. Samples were excluded if the positive control PHA was negative. The average of the DMSO negative control was subtracted per stimulation. The total spike-specific T cell response was defined by summing up the SFCs of the 2 peptide pools. To further assess the response rate for each cohort, participants who had a ≥ 2-fold increase in the number of spot-forming cells (SFCs) and ≥ 50 SFCs per 10^6^ PBMCs 28 days after vaccination compared to baseline were defined as responders. Responders were further subdivided into low (50 – 300 SFCs per 10^6^ PBMCs) and high (>300 SFCs per 10^6^ PBMCs) responders. This was based on experience in other infectious diseases using values in unvaccinated and uninfected healthy controls (50).

### Flow cytometry assay

PBMCs were thawed, washed and incubated at 37°C for 60 minutes in RPMI 1640 medium (Life Technologies, 21875-034) supplemented with human serum (Sigma, H3667, 10% v/v), penicillin-streptomycin (Life Technologies, 15140-122, 1% v/v) and benzonase nuclease (Sigma-Aldrich, 10104159001, 5 μg/mL). After washing, 0.5-1.5x10^6^ PBMCs were stimulated for 20 hours at 37°C with 15-mer peptide pools derived from the SARS-CoV-2 spike protein (JPT Peptide Technologies, 0.5 μg/mL) or equimolar amounts of DMSO (negative control). As technical control, 0.4x10^6^ PBMCs were stimulated with phorbol 12-myristate 13-acetate (Sigma, P8139-1MG, 50 ng/mL) and ionomycin (Sigma, I9657, 1 μg/mL). All conditions were cultured in the presence of GolgiPlug (BD, 555029, 1/1000) to prevent cytokine excretion. After stimulation cells were washed and stained for 20 minutes on ice with surface marker antibodies (**Supplementary Table 2**). Cells were washed and stained for 10 minutes on ice with LIVE/DEAD Fixable IR Dead Cell Stain Kit (Invitrogen, L10119, 1/400). Subsequently, cells were washed, fixed, and permeabilized using the Foxp3 Transcription Factor Staining Buffer Set (eBioscience, 00-5523-00) according to manufacturer’s protocol. Intracellular cytokine staining was performed for 20 minutes at room temperature with intracellular marker antibodies (**Supplementary Table 2**). Surface and intracellular marker staining was performed in the presence of Brilliant Staining Buffer Plus (BD, 566385) according to manufacturer’s protocol (BD). Samples were washed twice before acquisition. To account for day-to-day variation of the flow cytometer PMT voltages, a reference sample was used.

### Flow cytometer settings

All peptide stimulation samples were analyzed on the BD FACSymphony A5. The following 21-color instrument settings were used on the BD FACSymphony A5: blue laser (488 nm at 200 mW): FITC: 530/30BP, 505LP; BB700: 710/50BP, LP685. Red laser (637 nm at 140 mW): APC: 670/30BP, APC-R700: 690LP, 630/45BP; IRDye and APC-H7: 750LP, 780/60BP. Violet laser (405 nm at 100 mW): BV421: 431/28BP, 420LP; BV570: 586/15BP, 550LP; BV650: 661/11BP, 635LP; BV711: 711/25BP, 685LP. UV laser (355 nm at 75 mW): BUV395: 379/28BP; BUV563: 580/20BP, 550LP; BUV661: 670/25BP, 630LP; BUV737: 735/30BP, 690LP; BUV805: 819/44BP, 770LP. Yellow-green laser (561 nm at 150 mW): PE: 586/15BP; PE-eF610: 610/20BP, 600LP; PE-Cy7: 780/60BP, 750LP. Appropriate compensation controls were included in each analysis.

### Flow cytometry analysis

Spike-specific CD8 and CD4 T cell were identified using the following gating strategy using FlowJo (v.10.8.1) shown in **Supplementary Figure 2**: (1) selection of single-cell lymphocytes [forward scatter (FSC)-W/H low, side scatter (SSC)-W/H low, FSC/SSC-A] (2) selection of live (IRDye low-dim) cells negative for anti-CD14, anti-CD16, anti-CD19, (3) selection of anti-CD8 or anti-CD4 positive cells, and (4) selection of anti-TNF, anti-IFNγ, anti-IL-2, and/or anti-CCL4 positive CD8 and CD4 T cells, respectively. The frequency of spike-specific CD4 and CD8 T cells was assessed as followed: (1) Boolean gating of anti-TNF, anti-IFNγ, anti-IL-2, and anti-CCL4 positive cells was applied to determine the frequencies of 15 different spike-specific CD4/CD8 T cell populations in stimulated (SARS-CoV-2 spike protein) and unstimulated (DMSO) samples, (2) unspecific background (SARS-CoV-2 – DMSO) was subtracted for each of the 15 individual populations and negative values were set to 0, (3) the sum of individual spike-specific populations was used determine the total magnitude (percentage of total CD4/CD8 T cells) or proportion (percentage of total spike-specific CD4/CD8 T cell response) of monofunctional (producing one cytokine) or polyfunctional (producing ≥ 2 cytokines) spike-specific CD4/CD8 T cells. The quality of spike-specific CD4 and CD8 T cell responses was assessed using simplified presentation of incredibly complex evaluations (SPICE, v.6.1) (51).

### Statistical analysis

Differences in frequency of spike-specific T cells between two or three time points were assessed with a two-tailed Wilcoxon signed-rank test and non-parametric Friedman and Dunn’s multiple comparison test, respectively. Differences in frequency of spike-specific T cells between cohorts were assessed with a non-parametric Kruskal-Wallis and Dunn’s multiple comparison test. Differences in the functional phenotype of spike-specific T cell responses between cohorts were assessed with a permutation test with a multiple comparison test of 10.000 iterations. Differences in the proportion of individual functional spike-specific T cell populations between cohorts were assessed with an ordinary two-way ANOVA Tukey’s multiple comparisons test. Differences were considered significant if *P* < 0.05. Only significant *P* values are displayed. Data cut-off for all analyses was 16 April 2024. Statistical analysis was performed using Excel (v.16.83), PRISM 8 (v.8.4.0) and SPICE (v.6.1).

### High dimensional flow cytometry analysis

The phenotype of spike-specific CD4 and CD8 T cells was examined through Uniform Manifold Approximation and Projection (UMAP) analysis. To gain a comprehensive understanding of the spike-specific T cell responses in both the experimental and control settings, separate UMAP analyses were conducted for CD4 and CD8 T cells. Unstimulated and spike-stimulated samples from all participants were merged into a unified file with a unique identifier for each sample, and 5,000 cells were selected from each sample using an interval down-sampling method. The markers used in generating the UMAPs included CCR7, CD25, CD27, CD38, CD45RA, CD95, CD127 (only for CD4 T cells), HLA-DR, NKG2A (only for CD8 T cells), and Tim-3. The UMAPs were constructed following a scaling procedure to standardize the production values with the following settings: number of neighbors = 45, minimal distance = 0.1, and number of iterations = 3,000. Subsequently, the UMAPs were utilized to visualize the localization of cytokine-producing T cells, categorized as either monofunctional or polyfunctional. All analyses were conducted using FCS Express 7 software (*De Novo* Software in California, USA).

## Data Availability

The original contributions presented in the study are included in the article/**Supplementary Material**. Further inquiries can be directed to the corresponding author.
