# Supplementary material for: mRNA-1273 vaccination induces polyfunctional memory CD4 and CD8 T cell responses in patients with solid cancers undergoing immunotherapy or/and chemotherapy
Source: Front Immunol. 2024 Aug 27;15:1447555. doi: 10.3389/fimmu.2024.1447555 (PMC11385311; doi:10.3389/fimmu.2024.1447555)
Supplement: Supplementary file 1 [file DataSheet1.pdf]

Supplementary Information

mRNA-1273 vaccination induces polyfunctional memory CD4 and CD8 T cell responses in patients with solid cancers undergoing immunotherapy or/and chemotherapy

Anastasia Gangaev[1], Yannick van Sleen[2], Nicole Brandhorst[1], Kelly Hoefakker[1], Bimal Prajapati[2], Amrita Singh[2], Annemarie Boerma[2], Marieke van der Heiden[2], Sjoukje F. Oosting[3], Astrid A. M. van der Veldt[4], T. Jeroen N. Hiltermann[5], Corine H. GeurtsvanKessel[6], Anne-Marie C. Dingemans[7], Egbert F. Smit[8], Elisabeth G. E. de Vries[3,] John B. A. G. Haanen[1], Pia Kvistborg[1], Debbie van Baarle[2,9*]

[1]Division of Molecular Oncology and Immunology, The Netherlands Cancer Institute, Amsterdam, North Holland, 1066 CX, The Netherlands, [2]Department of Medical Microbiology and Infection Prevention, University Medical Centre Groningen, Groningen, Groningen, 9713 GZ, The Netherlands, [3]Department of Medical Oncology, University Medical Center Groningen, University of Groningen, Groningen, Groningen, 9713 GZ, The Netherlands, [4]Department of Medical Oncology and Radiology & Nuclear Medicine, Erasmus Medical Center Cancer Institute, Rotterdam, South Holland, 3015 GD, The Netherlands, [5]Department of Pulmonary Diseases, University Medical Centre Groningen, Groningen, Groningen, 9713 GZ, The Netherlands, [6]Department of Viroscience, Erasmus Medical Center Cancer Institute, University Medical Centre, Rotterdam, South Holland, 3015 GD, The Netherlands, [7]Department of Respiratory Medicine, Erasmus Medical Centre, Rotterdam, South Holland, 3015 GD, The Netherlands, [8]Department of Thoracic Oncology, The Netherlands Cancer Institute, Amsterdam, North Holland, 1066 CX, The Netherlands, [9]Centre for Infectious Disease Control, National Institute for Public Health and the Environment, Bilthoven, Utrecht, 3721 MA, The Netherlands, [*]Correspondence: Debbie van Baarle, mailing address: debbie.van.baarle@rivm.nl

Supplementary Figures

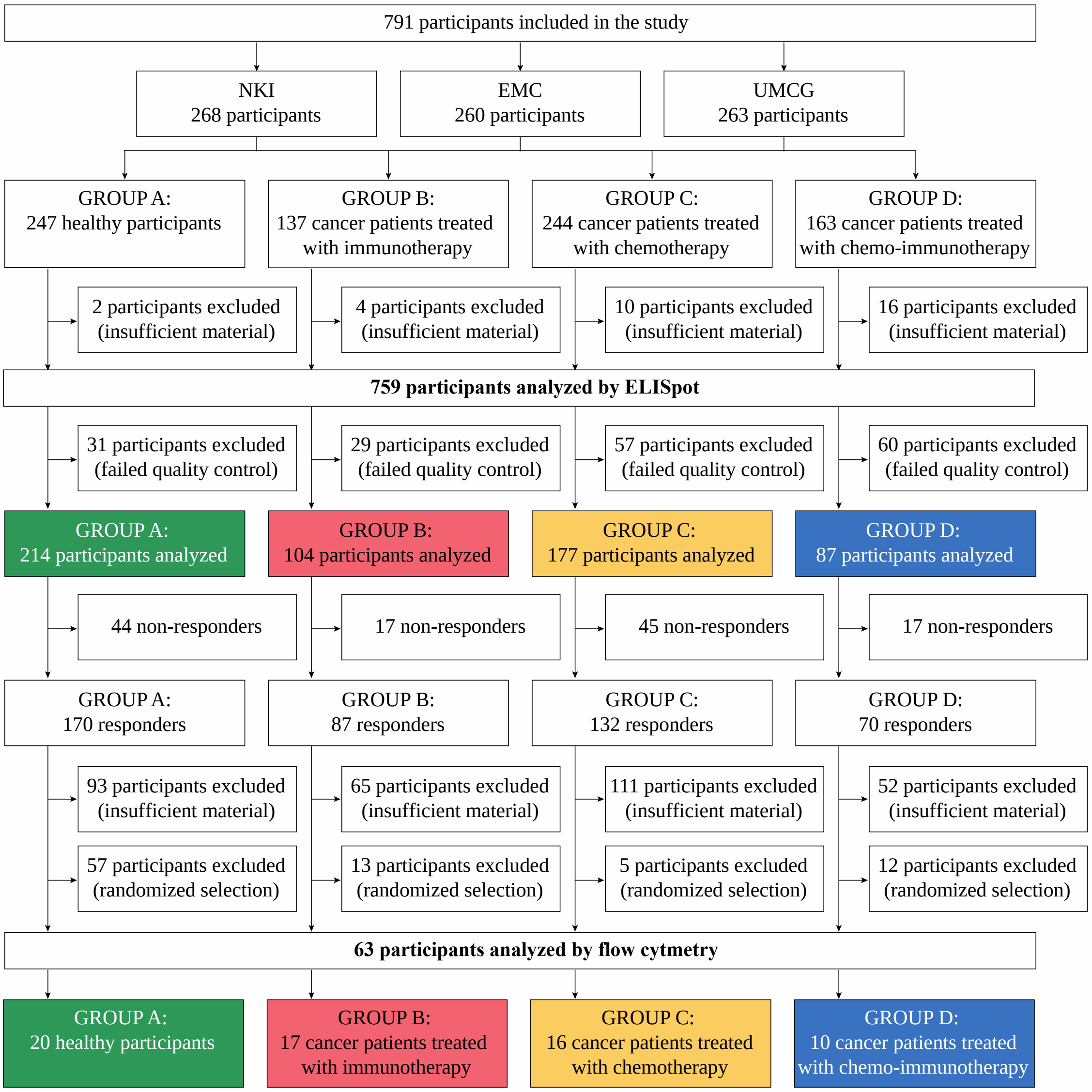

Supplementary Fig. 1: Flow diagram indicating numbers of included participants, selection steps and individual analyses performed in the study. NKI: Netherlands Cancer institute, UMCG: University Medical Centre in Groningen, EMC: Erasmus Medical Centre, CTRL: control, IT: immunotherapy, CT: chemotherapy, CT/IT: chemoimmunotherapy.

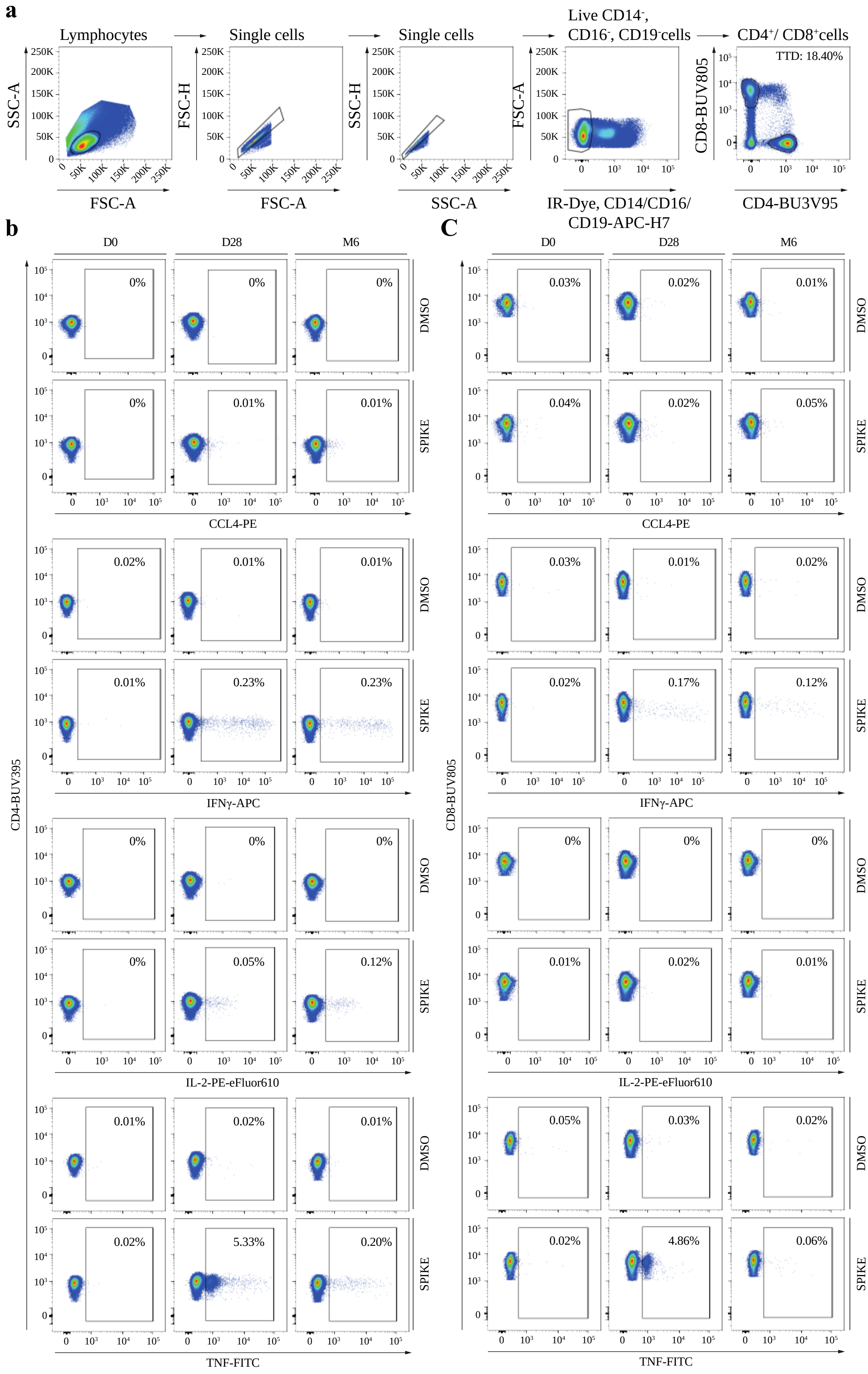
a
Lymphocytes
Single cells
Single cells
Live CD14-, CD16-, CD19-cells
CD4+/ CD8+cells
SSC-A
FSC-H
SSC-H
FSC-A
CD8-BUV805
TTD: 18.40%
FSC-A
FSC-A
SSC-A
IR-Dye, CD14/CD16/ CD19-APC-H7
CD4-BU3V95
b
C
D0
D28
M6
DMSO
SPIKE
CCL4-PE
IFNγ-APC
IL-2-PE-eFluor610
TNF-FITC
CD4-BUV395
CD8-BUV805

Supplementary Fig. 2: Identification of spike-specific T cell responses by flow cytometry. **a** Representative gating strategy used to identify live single CD4 and CD8 T cell populations. Representative gating strategy used to identify spike-specific **b** CD4 and **c** CD8 T cell responses based on production of CCL4, IFNγ, IL-2 and TNF after incubation with DMSO (negative control) or SARS-CoV-2 spike overlapping peptide pools. Percentages represent the frequency of cytokine producing cells of total CD4 or CD8 T cells.

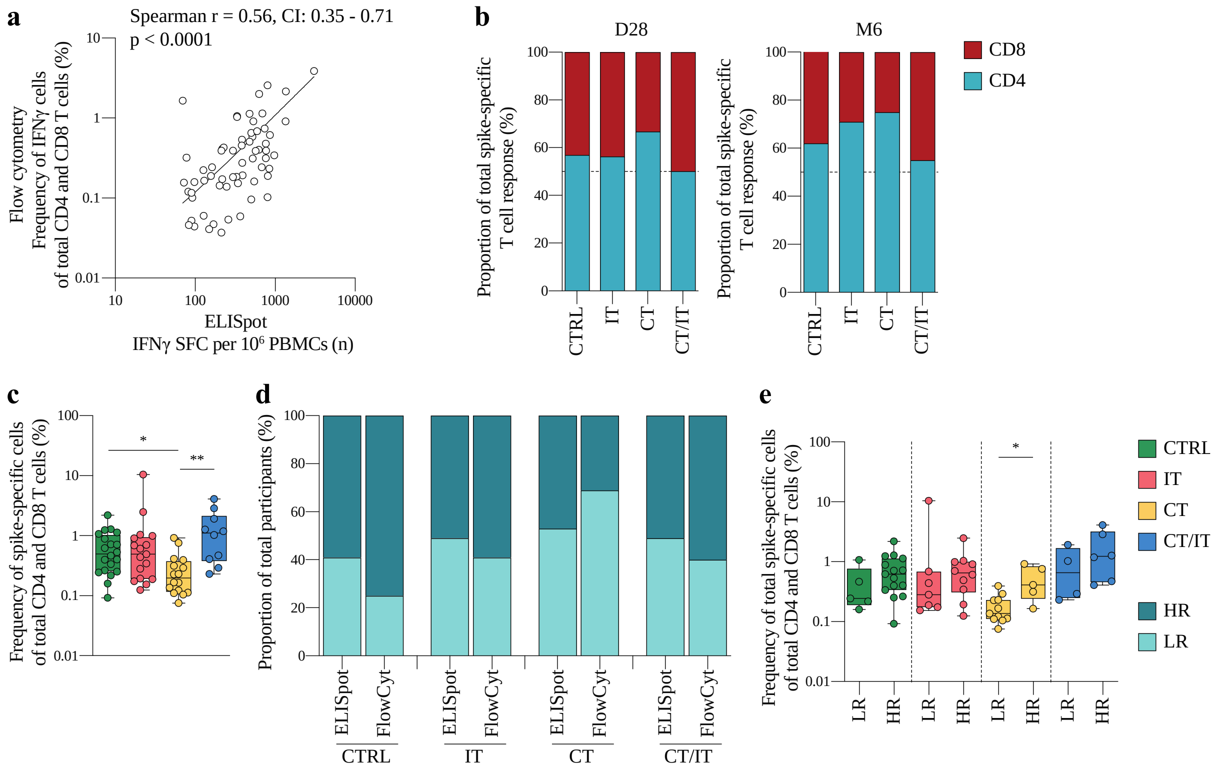

Supplementary Fig. 3: Spike-specific T cell responses identified by ELISpot and flow cytometry. **a** Correlation plot of SFC numbers measured by ELISpot and the total frequency of spike-specific CD4 and CD8 T cells detected by flow cytometry 28 days after the second vaccination (n = 63). **b** Median proportion of CD4 and CD8 T cells of the total spike-specific T cell response 28 days (left) and 6 months (right) after the second vaccination (CTRL n = 20, IT n = 17, CT n = 16, and CT/IT n = 10). **c** Total frequency of the spike-specific CD4 and CD8 T cell response measured by flow cytometry 28 days after the second vaccination (CTRL n = 20, IT n = 17, CT n = 16, and CT/IT n = 10). Box plots indicate the median (line), 25th and 75th percentile (box), min and max (whiskers), and individual data points (single circles). Statistical significance between cohorts was tested with a non-parametric Kruskal-Wallis and Dunn's multiple comparison test. **d** Bar graph illustrating the proportion of low and high responders defined by ELISpot and flow cytometry. **e** Differences in the total frequency of the spike-specific CD4 and CD8 T cell response measured by flow cytometry between low and high responders (CTRL: LR n = 5 / HR n = 15, IT: LR n = 7 / HR n = 10, CT: LR n = 12 / HR n = 5, and CT/IT: LR n = 4 / HR n = 6). Box plots indicate the median (line), 25th and 75th percentile (box), min and max (whiskers), and individual data points (single circles). Statistical significance was tested with non-parametric two-tailed Mann-Whitney *U*-test. * $P < 0.05$, ** $P < 0.01$. SFC: spot-forming cells, D28: day 28, CTRL: control, IT: immunotherapy, CT: chemotherapy, CT/IT: chemoimmunotherapy, HR: high responder, LR: low responder.

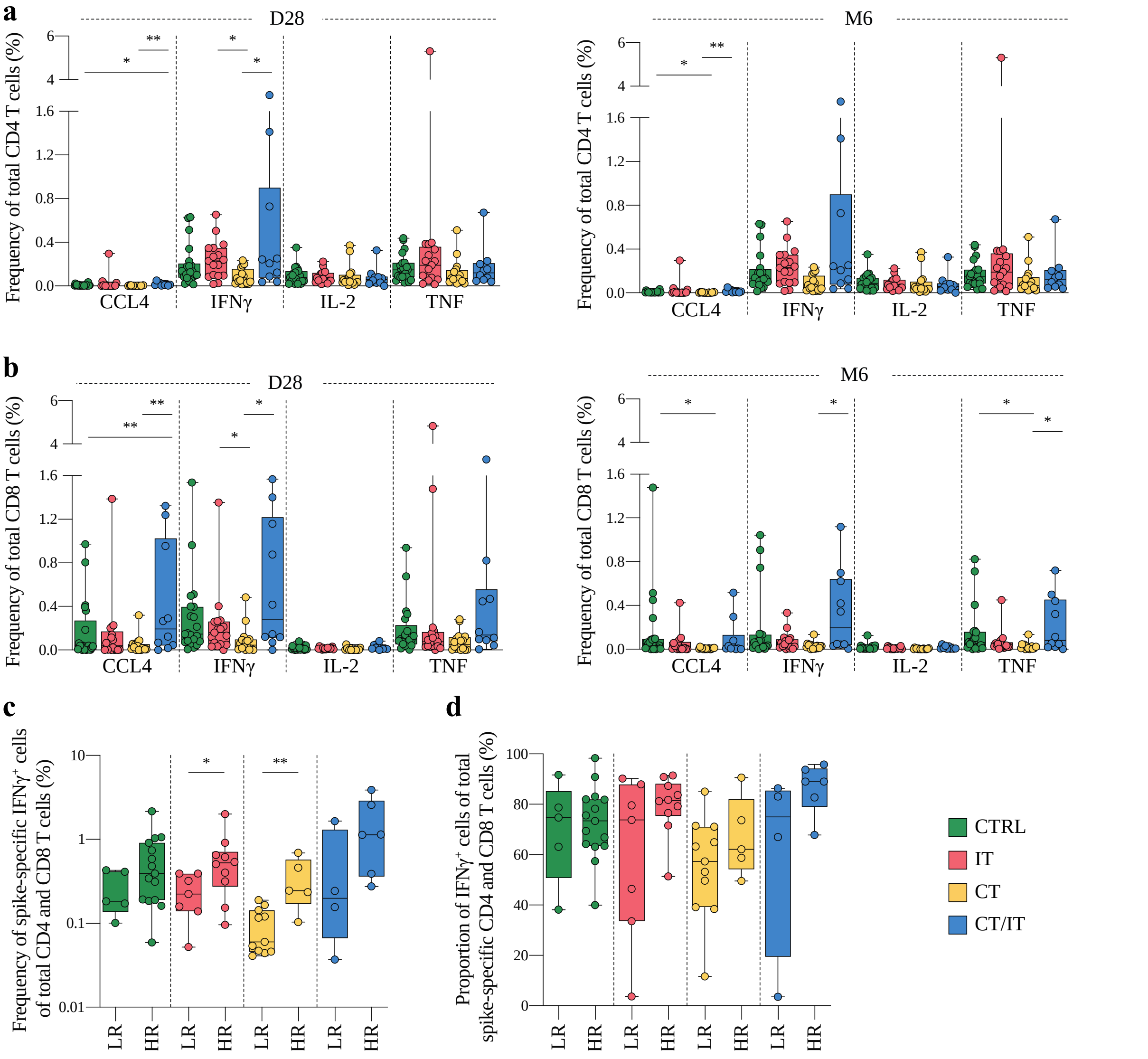

Supplementary Fig. 4: Frequency of spike-specific **a** CD4 and b CD8 T cells producing CCL4, IFNγ, IL-2 and TNF (CTRL n = 20, IT n = 17, CT n = 16, and CT/IT n = 10) 28 days and 6 months after the second vaccination. Box plots indicate the median (line), 25th and 75th percentile (box), min and max (whiskers), and individual points (single circles). Statistical significance between cohorts for each cytokine was tested with a non-parametric Kruskal-Wallis and Dunn's multiple comparison test. Differences in the frequency **c** and proportion **d** of IFNγ$^+$ CD4 and CD8 T cells measured by flow cytometry between low and high responders (CTRL: LR n = 5 / HR n = 15, IT: LR n = 7 / HR n = 10, CT: LR n = 12 / HR n = 5, and CT/IT: LR n = 4 / HR n = 6). Box plots indicate the median (line), 25th and 75th percentile (box), min and max (whiskers), and individual data points (single circles). Statistical significance was tested with non-parametric two-tailed Mann-Whitney *U*-test. * $P < 0.05$; ** $P < 0.01$; *** $P < 0.001$; **** $P < 0.0001$. D28: day 28, CTRL: control, IT: immunotherapy, CT: chemotherapy, CT/IT: chemoimmunotherapy, HR: high responder, LR: low responder.

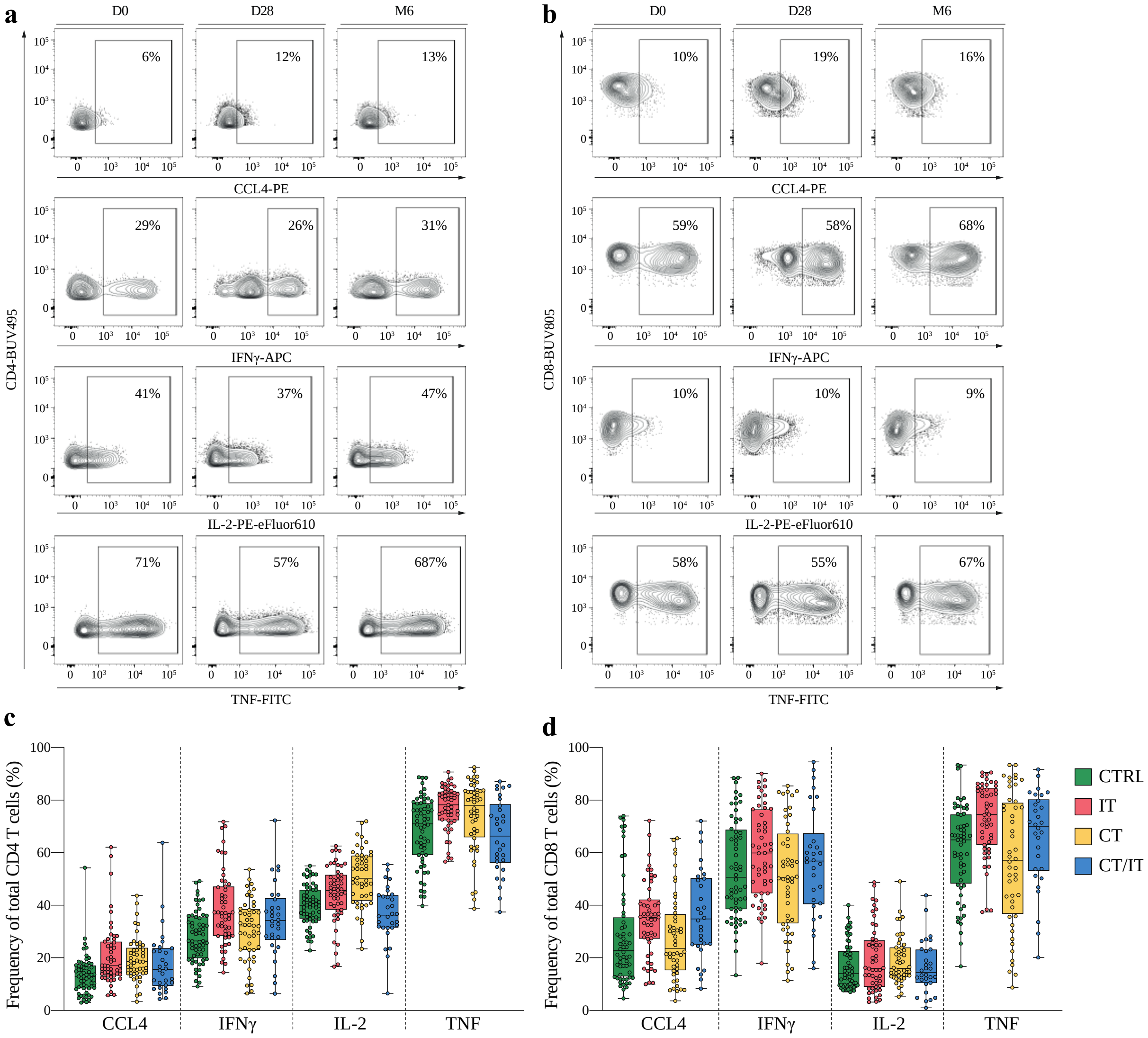

Supplementary Fig. 5: Technical controls using PMA/IO stimulation. Representative gating strategy used to assess production of CCL4, IFNγ, IL-2 and TNF after stimulation with PMA/IO in **a** CD4 and **b** CD8 T cells. Percentages represent the frequency of cytokine producing cells of total CD4 or CD8 T cells. Gating was based on the DMSO control (shown in Supplementary Fig. S2) and adjusted for the PMA/IO control if needed. Frequency of **c** CD4 and **d** CD8 T cells producting CCL4, IFNγ, IL-2 or TNF after stimulation with PMA/IO. Data from all analyzed time points is shown for each cohort (CTRL n = 60, IT n = 51, CT n = 48, and CT/IT n = 30). Box plots indicate the median (line), 25th and 75th percentile (box), min and max (whiskers), and individual points (single circles). CTRL: control, IT: immunotherapy, CT: chemotherapy, CT/IT: chemoimmunotherapy.

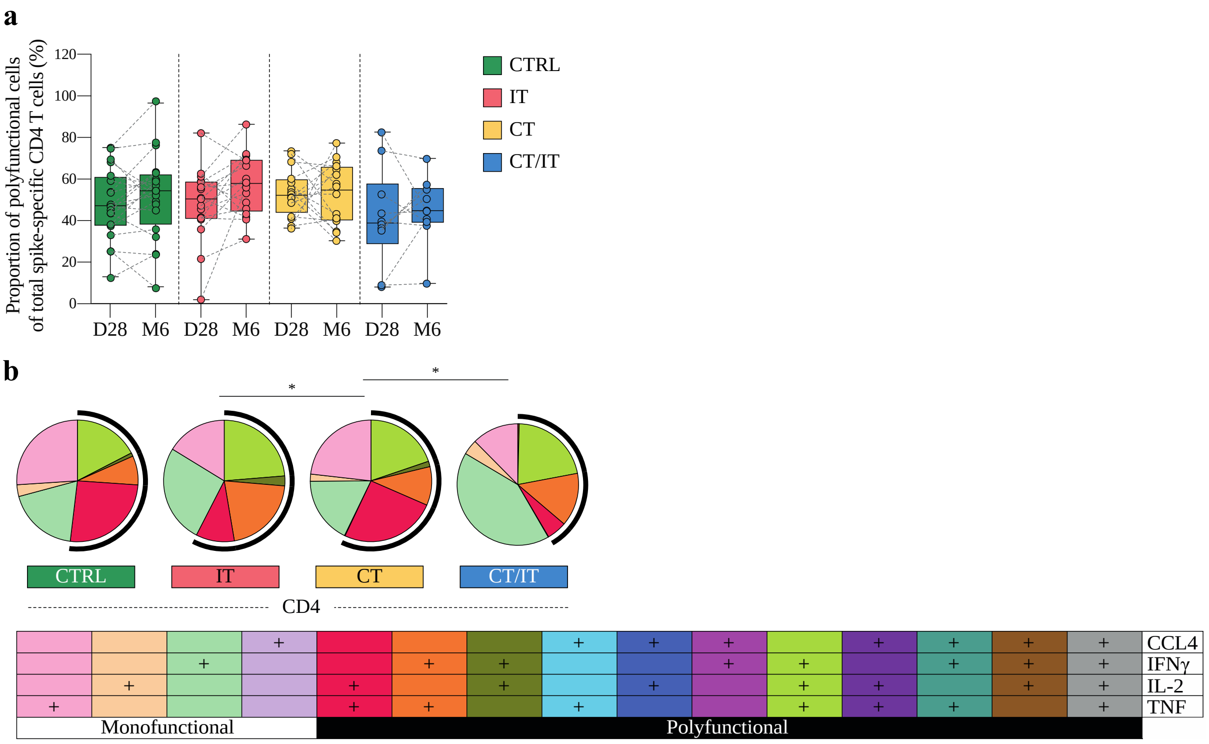

Supplementary Fig. 6: Functional quality of the spike-specific CD4 T cell response. **a** Proportion of polyfunctional cells of the total spike-specific CD4 T cell response 28 days and 6 months after the second vaccination (CTRL n = 20, IT n = 17, CT n = 16, and CT/IT n = 10). Box plots indicate the median (line), 25th and 75th percentile (box), 5th and 95th percentile (whiskers), and individual points (single circles). Statistical significance between cohorts for each individual time point was tested with a non-parametric Kruskal-Wallis and Dunn's multiple comparison test. **b** SPICE analysis of spike-specific CD4 T cell responses 6 months after the second vaccination (CTRL n = 20, IT n = 17, CT n = 16, and CT/IT n = 10). Pie charts represent the median proportion of individual spike-specific T cell populations of the total spike-specific CD4 T cell response. Pie chart colors represent the cytokine (co-)production patterns of individual spike-specific T cell populations. Arc legend indicates the proportion of polyfunctional spike-specific T cells of the total spike-specific CD4 T cell response. Statistical significance between cohorts was tested with a permutation test with a multiple comparison test of 10.000 iterations. * $P < 0.05$; ** $P < 0.01$; *** $P < 0.001$; **** $P < 0.0001$. D28: day 28, M6: month 6, CTRL: control, IT: immunotherapy, CT: chemotherapy, CT/IT: chemoimmunotherapy.

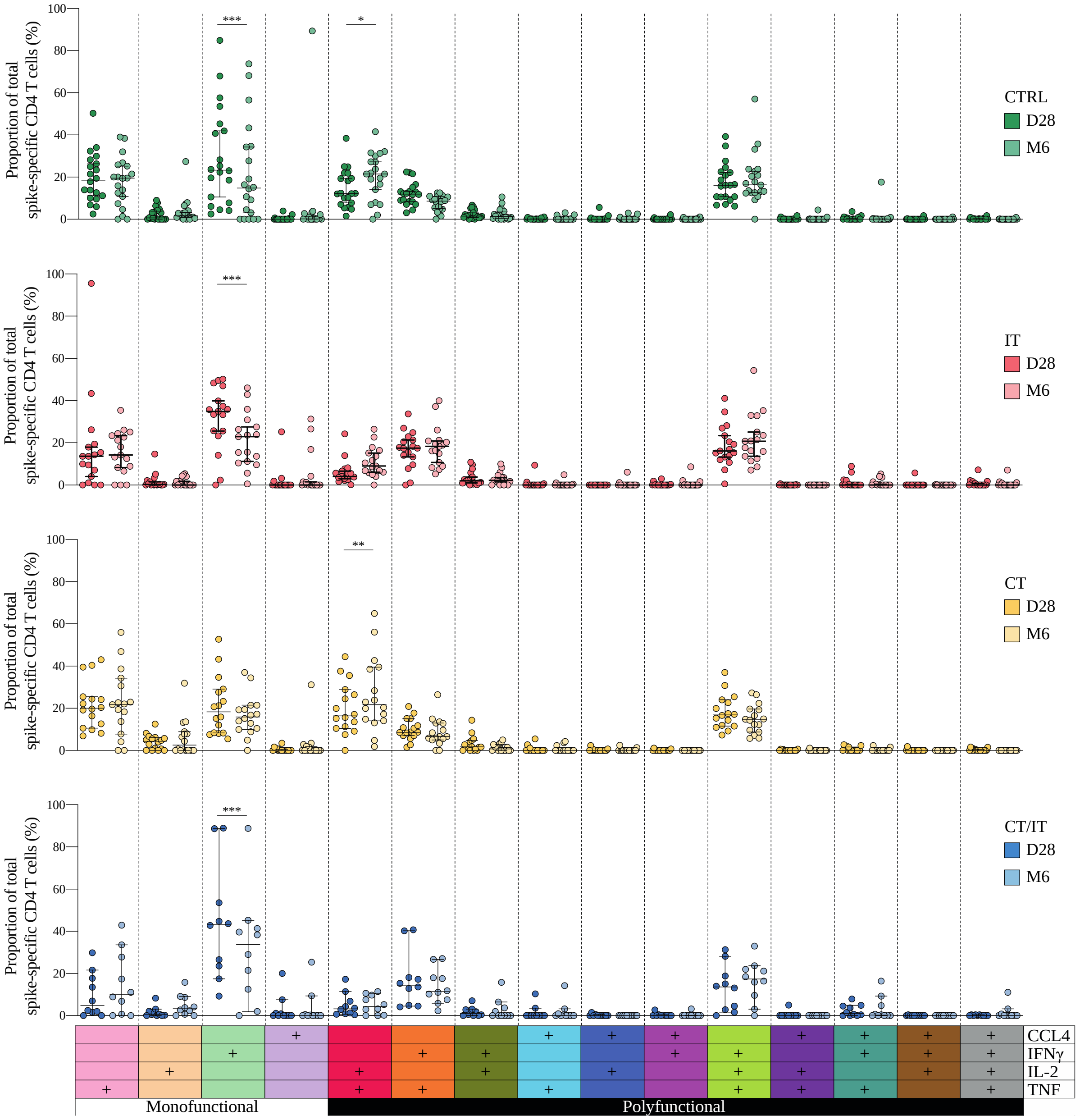

Supplementary Fig. 7: Proportion of the total spike-specific CD4 T cell response 28 days and 6 months after the second vaccination (CTRL n = 20, IT n = 17, CT n = 16, and CT/IT n = 10). Median (middle line), 95% confidence interval (whiskers) and individual points (single circles) are shown. Statistical significance between time points was tested with a RM two-way ANOVA Sidak's multiple comparisons test for each individual cohort. * $P < 0.05$; ** $P < 0.01$; *** $P < 0.001$. D28: day 28, M6: month 6, CTRL: control, IT: immunotherapy, CT: chemotherapy, CT/IT: chemoimmunotherapy.

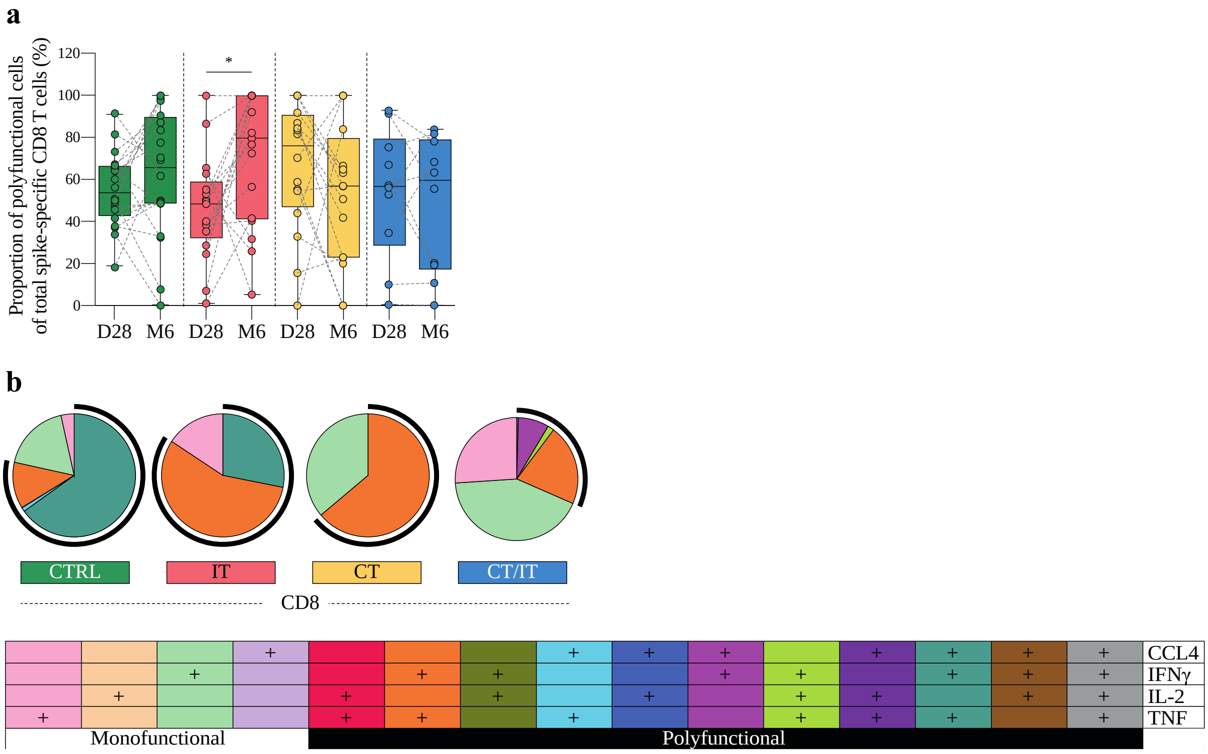

Supplementary Fig. 8: Functional quality of the spike-specific CD8 T cell response. **a** Proportion of polyfunctional cells of the total spike-specific CD8 T cell response 28 days and 6 months after the second vaccination (CTRL n = 20, IT n = 17, CT n = 16, and CT/IT n = 10). Box plots indicate the median (line), 25th and 75th percentile (box), 5th and 95th percentile (whiskers), and individual points (single circles). Statistical significance between cohorts for each individual time point was tested with a non-parametric Kruskal-Wallis and Dunn's multiple comparison test. **b** SPICE analysis of spike-specific CD8 T cell responses 6 months after the second vaccination (CTRL n = 20, IT n = 17, CT n = 16, and CT/IT n = 10). Pie charts represent the median proportion of individual spike-specific T cell populations of the total spike-specific CD8 T cell response. Pie chart colors represent the cytokine (co-)production patterns of individual spike-specific T cell populations. Arc legend indicates the proportion of polyfunctional spike-specific T cells of the total spike-specific CD8 T cell response. Statistical significance between cohorts was tested with a permutation test with a multiple comparison test of 10.000 iterations. * $P < 0.05$; ** $P < 0.01$; *** $P < 0.001$; **** $P < 0.0001$. D28: day 28, M6: month 6, CTRL: control, IT: immunotherapy, CT: chemotherapy, CT/IT: chemoimmunotherapy.

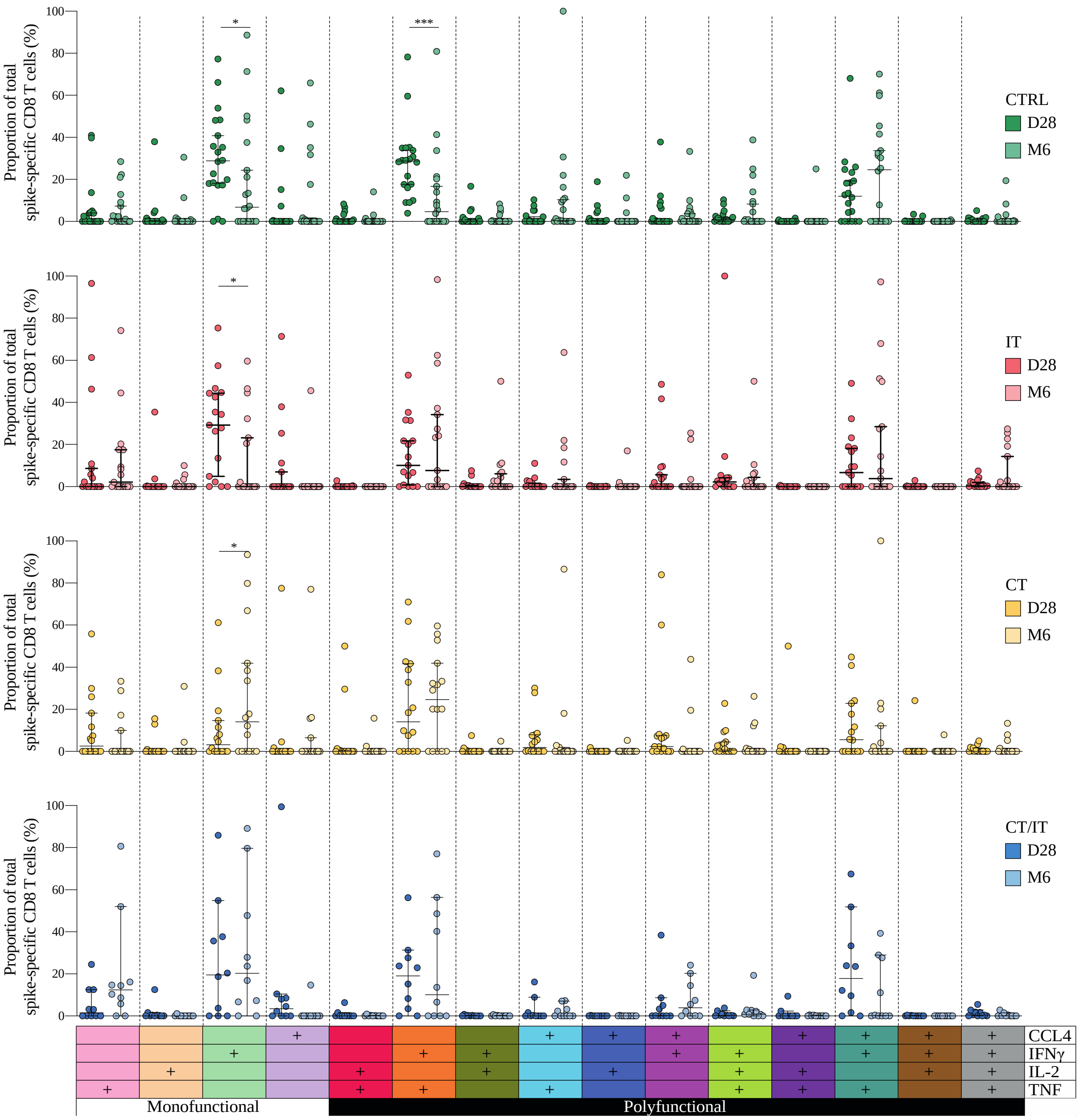

Supplementary Fig. 9: Proportion of the total spike-specific CD8 T cell response 28 days and 6 months after the second vaccination (CTRL n = 20, IT n = 17, CT n = 16, and CT/IT n = 10). Median (middle line), 95% confidence interval (whiskers) and individual points (single circles) are shown. Statistical significance between time points was tested with a RM two-way ANOVA Sidak's multiple comparisons test for each individual cohort. * $P < 0.05$; *** $P < 0.001$. D28: day 28, M6: month 6, CTRL: control, IT: immunotherapy, CT: chemotherapy, CT/IT: chemoimmunotherapy.

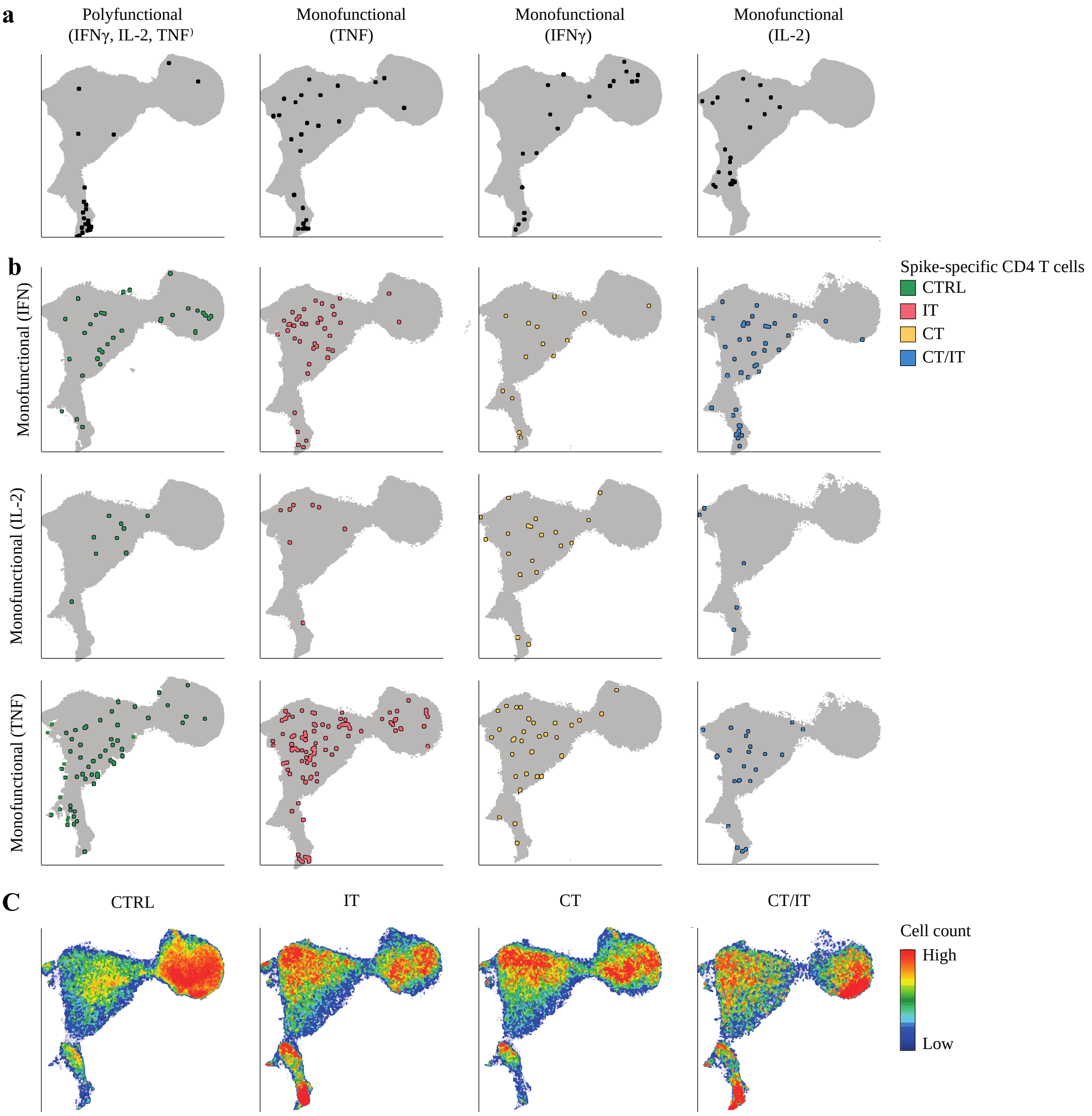

Supplementary Fig. 10: Phenotype of spike-specific T cells 28 days after the second vaccination. UMAPs were created based on merged files of unstimulated and stimulated CD4 T cells of each participant (total participants n = 63, cells per sample n = 5000). **a** UMAP localization of cytokine-producing CD4 T cells (black) after stimulation with DMSO (negative control) in CTRLs and patients with cancer treated with IT, CT, and CT/IT combined. Polyfunctional and monofunctional CD4 T cells were defined as cells producing ≥ 2 or only one of the analyzed cytokines (TNF, IFNγ, IL-2). **b** UMAP representation of monofunctional cytokine-producing CD4 T cells after stimulation with SARS-CoV-2 spike overlapping peptide pools for each cohort. **c** UMAP distribution of CD4 T cell counts in spike-stimulated samples for each cohort. CTRL: control, IT: immunotherapy, CT: chemotherapy, CT/IT: chemoimmunotherapy.

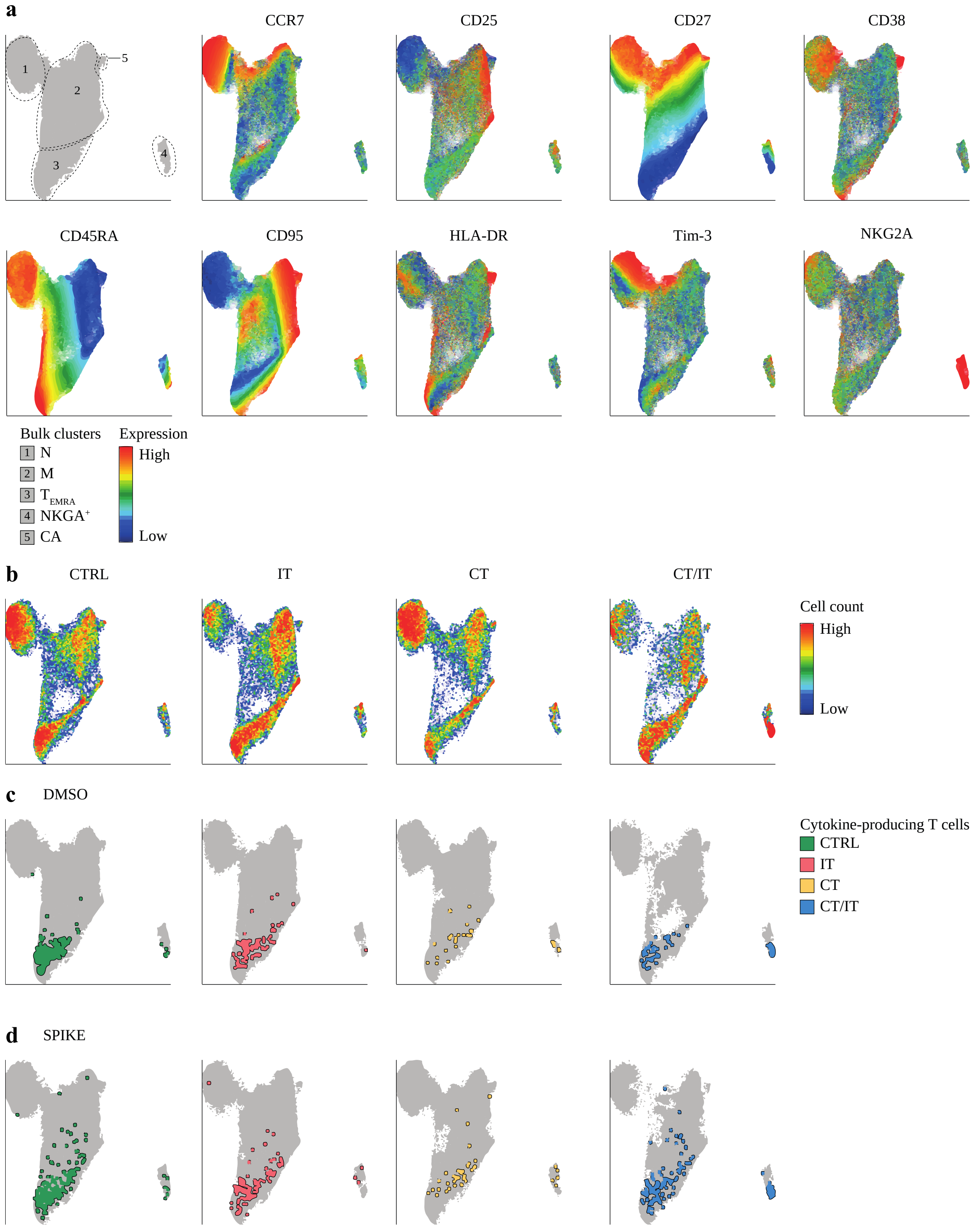

Supplementary Fig. 11: Phenotype of spike-specific CD8 T cells 28 days after the second vaccination. UMAPs were created based on merged files of unstimulated and stimulated CD8 T cells of each participant (total participants n = 63, cells per sample n = 5000). **a** UMAP depicting identified clusters and scaled production of analysed markers. UMAP depicting the localization of polyfunctional CD8 T cells after stimulation with **b** DMSO or with **c** SARS-

CoV-2 spike overlapping peptide pools for each cohort. Polyfunctional spike-specific CD8 T cells were defined as cells producing two or more of the analyzed cytokines (TNF, IFNγ, IL-2). **d** UMAP distribution of CD8 T cell counts in spike-stimulated samples for each cohort. UMAP: Uniform Manifold Approximation and Projection, N: naïve, M: memory, T_{EMRA}: terminally differentiated effector memory T cell, CA: chronically activated, CTRL: control, IT: immunotherapy, CT: chemotherapy, CT/IT: chemoimmunotherapy.

Supplementary Tables

Supplementary Table 1: Demographic and clinical characteristics of ELISpot responders and non-responders for individual cohorts. Detailed information for individual participants is provided in Supplementary Data 1.

	**CTRL** Individuals without cancer		**IT** Cancer patients treated with Immunotherapy		**CT** Cancer patients treated with Chemotherapy		**CT/IT** Cancer patients treated with Chemo-Immunotherapy	
**ELISpot response**	Non-responder	Responder	Non-responder	Responder	Non-responder	Responder	Non-responder	Responder
**Total number of participants (n)**	44	170	17	87	45	132	17	70
**Age, median (range)**	62 (20-84)	66 (20-87)	68 (50-82)	66 (29-83)	61 (19-76)	59 (19-76)	63 (48-75)	62 (33-82)
**Gender, n (%)**								
Female	22 (50%)	81 (48%)	3 (18%)	32 (37%)	26 (58%)	83 (63%)	8 (47%)	38 (54%)
Male	22 (50%)	89 (52%)	14 (82%)	55 (63%)	19 (42%)	49 (37%)	9 (53%)	32 (46%)
**WHO performance status, n (%)**								
0	44 (100%)	170 (100%)	7 (41%)	66 (76%)	24 (53%)	80 (61%)	7 (41%)	29 (41%)
1	0 (0%)	0 (0%)	10 (59%)	21 (24%)	20 (44%)	50 (38%)	10 (59%)	33 (47%)
2	0 (0%)	0 (0%)	0 (0%)	0 (0%)	1 (2%)	2 (2%)	0 (0%)	8 (11%)
Unknown	0 (0%)	0 (0%)	0 (0%)	0 (0%)	0 (0%)	0 (0%)	0 (0%)	0 (0%)
**Tumor stage, n (%)**								
I	n/a	n/a	1 (6%)	1 (1%)	5 (11%)	8 (6%)	0 (0%)	0 (0%)
II	n/a	n/a	1 (6%)	0 (0%)	4 (9%)	27 (20%)	0 (0%)	0 (0%)
III	n/a	n/a	2 (12%)	25 (29%)	7 (16%)	32 (24%)	0 (0%)	7 (10%)
IV	n/a	n/a	13 (76%)	61 (70%)	29 (64%)	64 (48%)	17 (100%)	63 (90%)
Unknown	n/a	n/a	0 (0%)	0 (0%)	0 (0%)	1 (1%)	0 (0%)	(0%)
**Treatment intent, n (%)**								
Curative	n/a	n/a	0 (0%)	32 (37%)	22 (49%)	74 (56%)	1 (6%)	11 (16%)
Non-curative	n/a	n/a	17 (100%)	55 (63%)	23 (51%)	58 (44%)	16 (94%)	59 (84%)
**Primary tumor localisation, n (%)**								
Bone, articular cartilage and soft tissues	n/a	n/a	0 (0%)	1 (1%)	2 (4%)	5 (4%)	0 (0%)	0 (0%)
Breast	n/a	n/a	0 (0%)	0 (0%)	12 (27%)	44 (33%)	0 (0%)	0 (0%)
Central nervous system	n/a	n/a	0 (0%)	0 (0%)	0 (0%)	7 (5%)	0 (0%)	0 (0%)
Digestive tract	n/a	n/a	0 (0%)	4 (5%)	15 (33%)	36 (27%)	0 (0%)	0 (0%)
Endocrine glands	n/a	n/a	0 (0%)	0 (0%)	2 (4%)	1 (1%)	0 (0%)	0 (0%)
Female genital organs	n/a	n/a	0 (0%)	0 (0%)	2 (4%)	13 (10%)	0 (0%)	0 (0%)
Head and Neck	n/a	n/a	0 (0%)	1 (1%)	2 (4%)	3 (2%)	0 (0%)	1 (1%)
Male genital organs	n/a	n/a	0 (0%)	0 (0%)	7 (16%)	5 (4%)	0 (0%)	0 (0%)
Respiratory tract	n/a	n/a	4 (24%)	14 (16%)	1 (2%)	11 (8%)	17 (100%)	69 (99%)
Skin	n/a	n/a	6 (35%)	49 (56%)	0 (0%)	0 (0%)	0 (0%)	0 (0%)
Urinary tract	n/a	n/a	7 (41%)	17 (20%)	2 (4%)	7 (5%)	0 (0%)	0 (0%)
Other/ unspecified sites	n/a	n/a	0 (0%)	1 (1%)	0 (0%)	0 (0%)	0 (0%)	0 (0%)

Supplementary Table 2: Antibodies used in this study. N/A: not applicable.

Antibody	Fluorochrome	Clone	Company	Cat. No.	Lot. No.	Dilution
CCL4	PE	D21-1351	BD	550078	1138594	100
CCR7	BV711	150503	BD	566602	2028505	133
CD127	BV570	A019D5	BioLegend	351308	B326880	50
CD14	APC-H7	MφP9	BD	560180	1324108	100
CD16	APC-H7	3G8	BD	560195	1077719	100
CD19	APC-H7	SJ25C1	BD	560177	1152663	100
CD25	APC-R700	2A3	BD	565106	1246164	400
CD27	BB700	M-T271	BD	566449	1229074	100
CD38	BV421	HIT2	BD	562444	1250192	200
CD4	BUV395	SK3	BD	563550	1040419	50
CD45RA	BUV563	HI100	BD	612926	1078217	400
CD8	BUV805	SK1	BD	612889	1296376	50
CD95	BUV737	DX2	BD	612790	2010599	50
HLA-DR	BUV661	G46-6	BD	612980	1210169	400
IFN-γ	APC	B27	BD	554702	1284406	400
IL-2	PE-eFluor610	MQ1-17H12	Thermofisher	61-7029-42	2162729	100
LIVE/DEAD Near IR-Dye	APC-H7	N/A	Thermofisher	L10119	N/A	800
NKG2A	PE-Cy7	Z199	Beckman	B10246	200068	100
Tim-3	BV650	7D3	BD	565564	1083787	200
TNF	FITC	MAb11	BD	554512	1026907	50
